# Epidemiological models in health misinformation propagation of Mpox conversations

**DOI:** 10.1371/journal.pdig.0001772

**Published:** 2026-11-13

**Authors:** Udoka Duruaku, Tawakalit Omolabake Agboola, Oluwaseun Ajao

**Affiliations:** School of Computing and Mathematics, Manchester Metropolitan University, Manchester, United Kingdom; Massachusetts Institute of Technology, UNITED STATES OF AMERICA

## Abstract

The rapid dissemination of health misinformation through digital platforms poses significant challenges to public health communication and response strategies. The spread of misinformation can be likened to the spread of diseases within communities during an epidemic. This study addresses the question around how the classical SIR (Susceptible-Infectious-Recovered) and adapted SEIR (Susceptible-Exposed-Infectious-Recovered) epidemiological models compare in their abilities to capture the temporal dynamics of health misinformation spread on social media platforms during disease outbreaks. Using the 2022 Mpox outbreak as a case study, we analyse a publicly available dataset of 5,787 tweets collected between 7–13 July 2022, with the vast majority posted on a single day (9 July 2022). Given the narrow temporal window and concentrated activity in this dataset, we frame our study as a *proof-of-concept* illustrating how compartmental models can be adapted to short-horizon social media bursts, rather than a definitive epidemiological characterisation of Mpox misinformation. Within this scope, our comparative analysis shows that the SIR model effectively captures rapid initial dissemination patterns characteristic of viral misinformation spread, whilst the SEIR model more accurately represents the latency period between exposure and active sharing behaviour observed in social media contexts. We report explicit parameter values (β, γ, σ), discuss their sensitivity and identifiability limits under the short observation window, and highlight the restrictions imposed by the homogeneous-mixing assumption. These findings contribute methodological insights for compartmental model selection in misinformation research and motivate future work with larger, longitudinally collected datasets.

## Author summary

Health misinformation, meaning false or misleading claims about diseases, treatments or outbreaks, can spread through social media much like an infectious disease spreads through a population. In this study we borrow mathematical models originally designed for tracking epidemics (SIR and SEIR models) and apply them to the spread of misinformation about Mpox (formerly monkeypox) on Twitter during July 2022. We treat each social media user as a person who can be “susceptible” to misinformation, “exposed” (having seen it but not yet shared it), “infectious” (actively sharing it), or “recovered” (no longer sharing). Using a publicly available dataset of roughly 5,800 tweets, we show that a model including an explicit “exposed” stage better reflects real social media behaviour, where people typically see a post several times before deciding to share it. Because the dataset is small and concentrated on a single day, we present this work as a proof-of-concept rather than a definitive analysis. Our findings suggest that these epidemic-style models can be a useful lens for studying how health misinformation moves through online communities, and they highlight where larger datasets and more realistic models are needed.

## 1 Introduction

### 1.1 Background and motivation

The proliferation of misinformation through digital channels represents a critical challenge to public health and societal well-being [1]. False or misleading information can undermine trust in institutions, hinder effective public health responses, and directly threaten population health during disease outbreaks [2]. The COVID-19 pandemic starkly illustrated how health misinformation can spread as rapidly as the virus itself, creating what the World Health Organization termed an “infodemic” [3]. Understanding the mechanisms underlying misinformation propagation is therefore essential for developing effective countermeasures [4].

Epidemiological models have long been used to understand disease transmission dynamics [5,6], and researchers have increasingly adapted these frameworks to study information diffusion in social networks [7]. The conceptual parallel is intuitive: just as infectious diseases spread through contact between susceptible and infected individuals, information (both accurate and inaccurate) spreads through communication between network members [8]. However, significant questions remain about which model structures best capture the unique characteristics of information spread compared to biological contagion [9].

### 1.2 Theoretical foundations

#### Classical epidemiological models.

The foundational SIR (Susceptible-Infectious-Recovered) model, introduced by Kermack and McKendrick in 1927 [5], divides a population into three compartments based on disease status. Susceptible individuals can become infected through contact with infectious individuals, and infectious individuals eventually recover (gaining immunity) or are removed from transmission [10]. The model is governed by the differential equations:


$$\begin{array}{cc} \frac{dS}{dt} & =-\beta SI \end{array}$$ (1)

$$\begin{array}{cc} \frac{dI}{dt} & =\beta SI-\gamma I \end{array}$$ (2)

$$\begin{array}{cc} \frac{dR}{dt} & =\gamma I \end{array}$$ (3)

where *S*, *I*, and *R* represent the proportions of susceptible, infectious, and recovered individuals, β is the transmission rate, and γ is the recovery rate [11,12].

The SEIR model extends this framework by introducing an Exposed (E) compartment, representing individuals who have been infected but are not yet infectious [6]. This latency period, characteristic of many infectious diseases, is governed by the rate parameter σ:


$$\begin{array}{cc} \frac{dS}{dt} & =-\beta SI \end{array}$$ (4)

$$\begin{array}{cc} \frac{dE}{dt} & =\beta SI-\sigma E \end{array}$$ (5)

$$\begin{array}{cc} \frac{dI}{dt} & =\sigma E-\gamma I \end{array}$$ (6)

$$\begin{array}{cc} \frac{dR}{dt} & =\gamma I \end{array}$$ (7)

These compartmental models have been extensively studied and their mathematical properties are well-understood [10,12].

#### Rumor spreading models.

Parallel to disease modeling, researchers have developed specific models for rumor propagation. The Maki-Thompson model [13], a canonical framework in rumor theory, introduces the concept of “stiflers”—individuals who have heard the rumor but choose not to spread it, analogous to the recovered compartment. Daley and Kendall’s earlier model [14] similarly conceptualized rumor spread through population compartments, establishing the theoretical foundation for applying epidemic models to information diffusion.

More recent work has extended these classical models to account for network structure [15], forgetting mechanisms [16], and varying credibility perceptions [17]. Zhao et al. [18] specifically adapted the SIR framework for social media contexts, while other researchers have developed multi-compartment models incorporating skepticism and awareness states [19,20].

#### Psychology of misinformation.

From a psychological perspective, misinformation spread is influenced by cognitive biases, motivated reasoning, and social identity factors [4]. The continued influence effect demonstrates that corrections often fail to eliminate misinformation’s impact on beliefs and decision-making [2]. Heuristic processing and confirmation bias can lead individuals to accept and share false information that aligns with their preexisting beliefs [21,22]. Understanding these psychological mechanisms is crucial for interpreting the behavioural patterns captured by mathematical models.

### 1.3 Research gap and objective

Despite extensive literature on both epidemiological modeling and information diffusion, several critical gaps remain. First, while many studies have applied SIR or SEIR models to information spread [23–25], few have rigorously compared these models’ relative performance in capturing real-world misinformation dynamics during actual health crises [26]. Second, the translation of biological parameters (transmission, latency, recovery) to information contexts remains theoretically underdeveloped [8]. Third, most studies do not adequately address the fundamental differences between disease and information transmission, particularly regarding network heterogeneity and conscious decision-making in sharing behaviour [9].

**Research question:** This study addresses the following focused research question: *How do the SIR and SEIR epidemiological models compare in their ability to capture the temporal dynamics and key parameters of health misinformation spread on Twitter during the 2022 MPox outbreak?*

**Specific Objectives:**

1. To implement and parametrize both SIR and SEIR models using empirical MPox misinformation data from Twitter [27]
2. To compare model predictions against observed temporal patterns of misinformation tweet volume
3. To identify which model structure better accounts for the latency between exposure and active sharing in social media contexts
4. To evaluate the sensitivity of model parameters to initial conditions and their interpretability in information diffusion contexts

This focused comparative approach contributes methodological insights for model selection in misinformation research and provides empirical evidence for the importance of latency periods in social media information cascades.

## 2 Methodology

### 2.1 Data source, provenance and scope

We analysed the publicly available “Monkeypox Tweets” dataset compiled by Stephen Crone and hosted on Kaggle [27]. The dataset was originally curated and released as an educational resource for data-science practitioners interested in short-form text classification and social media analytics. We describe its provenance, fields and temporal scope in detail and explicitly discuss the implications of its limitations for our findings.

#### Provenance.

The dataset was assembled by the Kaggle contributor by querying Twitter’s public API during the period of heightened international attention to Mpox cases in summer 2022. The maintainer released the data under an open licence for non-commercial, educational use. Tweets were retrieved using keyword-based filtering (“monkeypox” and close variants) and each tweet was subsequently labelled by the contributor with a binary misinformation flag through manual inspection against contemporaneous public health guidance (e.g., WHO and CDC communications on Mpox). Because labelling was performed by the dataset author rather than a panel of trained fact-checkers, label noise is a known limitation and we treat results as indicative rather than definitive.

#### Temporal scope.

The dataset contains 5,787 tweets with tweet_created timestamps spanning 7–13 July 2022. Importantly, the distribution of tweets across these dates is highly non-uniform: the overwhelming majority of records are concentrated on 9 July 2022, with only sparse activity on surrounding dates. Effectively, the dataset provides dense coverage of a single burst day embedded in a seven-day window. This is a central constraint on our analysis and is discussed further in Section 4.5.

#### Fields.

The dataset provides 16 features per tweet: tweet_created (timestamp), tweet (text), user_is_verified, user_has_url, user_description, user_created_at, engagement metrics (retweet_count, reply_count, like_count, quote_count), user-level metrics (followers_count, following_count, tweet_count, listed_count), user_location, and a binary misinformation label. Table 1 summarises these fields and their role in our modelling.

**Table 1:** Fields in the Crone Mpox Twitter dataset [27] and their role in the present analysis.

Field	Type	Description / use in this study
tweet_created	datetime	Tweet post time; drives temporal analysis and model fitting.
tweet	text	Tweet content; retained but not used for feature engineering in this study.
user_is_verified	boolean	Platform verification flag; feature for Bayesian network and classifier.
user_has_url	boolean	Whether the profile lists a URL.
user_description	text	User biography; not modelled here.
user_created_at	datetime	Derived: account age as predictor of sharing behaviour.
retweet_count	integer	Engagement metric; feature and outcome proxy.
reply_count	integer	Engagement metric.
like_count	integer	Engagement metric.
quote_count	integer	Engagement metric.
followers_count	integer	Audience reach; classifier feature.
following_count	integer	Following cardinality.
tweet_count	integer	Lifetime tweets by the user; classifier feature.
listed_count	integer	Number of public lists the user appears on.
user_location	text	Free-text location; summarised descriptively only.
misinformation	binary	Target label (1 = misinformation, 0 = other); assigned by dataset author.

#### Representativeness.

The dataset is *not* a census of Mpox-related discourse on Twitter during July 2022. It represents a convenience sample obtained under the constraints of public API access at the time of collection and is dominated by a single high-volume day. We therefore explicitly frame the present study as a *proof-of-concept* demonstration of how compartmental models can be fit to short, bursty social media records; we do not claim generalisable epidemiological parameters for Mpox misinformation at large. Collection of a larger, longitudinally balanced dataset, either through academic Twitter API access or alternative platforms, is identified as a priority for future work.

### 2.2 Data preprocessing

Following established practices in social media analysis [28,29], we converted temporal features from object to datetime format to enable time-series analysis. We retained all tweets regardless of verification status to capture the full spectrum of information propagation, as prior research indicates that misinformation spreads through both verified and unverified accounts [9].

#### Label coding: Misinformation vs. other.

The source dataset contains a binary label distinguishing misinformation from all other content. We preserved this binary coding rather than constructing a three-way “misinformation / verified-good-information / neutral” scheme for two reasons. First, the dataset author did not separate “good information” from “other” labels in a way that would support a reliable trichotomy. Second, and more importantly, the compartmental models we fit treat “exposure” as exposure to the modelled pathogen (here, misinformation), so the relevant dichotomy is whether a user encounters/shares misinformation or not. We note explicitly that this simplification departs from the lived experience of social media users, who are simultaneously exposed to both accurate and inaccurate content; this is an important disanalogy with biological contagion (where an individual is either exposed or not), and it is one reason why our compartmental fits should be interpreted as descriptive rather than mechanistically faithful. Extensions to competing-strain or two-pathogen models that explicitly track exposure to good information alongside misinformation are an important direction for future work.

### 2.3 Model implementation

#### SIR model specification.

We implemented the discrete-time SIR model [11] with the following adaptations for Twitter data:

- **Susceptible (S):** Users who have not yet been exposed to MPox misinformation tweets
- **Infectious (I):** Users actively sharing/retweeting misinformation content
- **Recovered (R):** Users who have seen misinformation but stopped engaging with or sharing it

Initial conditions were set based on empirical data from July 7, 2022:

- *S*_0_ = 5785 (total dataset size minus initial misinformation tweets)
- *I*_0_ = 2 (initial misinformation tweets observed at *t* = 0)
- *R*_0_ = 0 (no recovered individuals at outbreak start)

The transmission parameter β was estimated from the empirical growth rate during the first 24 hours, and the recovery parameter γ was calibrated to match the observed decline phase [26]. We used a discrete time step of 5 hours to match the temporal resolution of significant changes in tweet volume.

#### SEIR model specification.

The SEIR model incorporates an additional Exposed (E) compartment representing users who have encountered misinformation but have not yet actively shared it [6]. This latency period is particularly relevant for social media contexts where users often observe content before deciding whether to engage [30].

Initial conditions for SEIR:

- *S*_0_ = 5775 (slightly reduced to account for exposed individuals)
- *E*_0_ = 10 (users estimated to have seen but not yet shared misinformation)
- *I*_0_ = 2 (actively sharing misinformation)
- *R*_0_ = 0

The latency rate parameter σ represents the rate at which exposed users transition to active sharing, estimated from the observed delay between tweet views (inferred from engagement metrics) and retweets [31].

#### Parameter estimation and values.

Parameters were estimated using a combination of direct calculation from empirical data and least-squares fitting to observed time series [32]. We used Python’s SciPy optimisation library to minimise the sum of squared errors between model predictions and observed misinformation tweet counts. Table 2 reports the specific parameter values used in each simulation together with their interpretation. All simulations use a discrete time step of 5 hours, chosen to align with the temporal resolution of meaningful volume changes in the dataset (which is dominated by a single burst day). The recovery time 1/γ should be interpreted as the mean time a user remains in the “actively sharing” compartment before ceasing engagement with the topic, here on the order of hours, reflecting Twitter’s fast decay of individual posts in timelines rather than a biologically meaningful recovery period.

**Table 2:** Parameter values for the SIR and SEIR simulations, fit by least squares to the empirical tweet volume time series. β is per susceptible–infectious contact per 5-hour step given *N* = 5,787; the effective basic reproduction number is $R_{0}=\beta N/\gamma \approx 1.7$. Mean infectious duration is 1/γ≈50 h; mean SEIR latency is 1/σ≈25 h.

Param.	Meaning	SIR	SEIR
β	Transmission rate (per contact, per step)	$3.0\times 10^{-4}$	$3.0\times 10^{-4}$
σ	Progression rate E→I	–	0.20 step^–1^
γ	Recovery rate I→R	0.10 step^–1^	0.10 step^–1^
Δt	Time step	5 h	5 h
*S*_0_	Initial susceptible	5,785	5,775
*E*_0_	Initial exposed	–	10
*I*_0_	Initial infectious	2	2
$R_{0}^{\;(\text{init})}$	Initial recovered	0	0
Horizon	Simulated duration	1,3,5,7 d	7 d

Sensitivity analysis was conducted by varying each parameter within plausible ranges based on prior literature [15,33]; results are summarised in S1 Appendix.

### 2.4 Bayesian network analysis

To complement the temporal dynamics analysis, we employed Bayesian network modeling to identify probabilistic dependencies between tweet features and misinformation propagation [34]. This approach differs fundamentally from social network analysis; Bayesian networks represent causal probabilistic relationships between variables rather than interpersonal connections in a social graph [35].

We constructed the network using structure learning algorithms implemented in pgmpy (Python library for probabilistic graphical models), with misinformation status as the target variable. Parent nodes were selected from tweet features including user verification, engagement metrics (retweet, reply, quote counts), and structural measures. The resulting conditional probability distributions quantify how these factors influence misinformation likelihood, providing interpretable insights beyond the temporal models [34].

### 2.5 Machine learning classification

To assess the predictability of misinformation based on tweet and user features, we implemented ensemble classification methods [36]. We split the dataset 80–20 into training and test sets, applying standard scaling to numerical features. Three algorithms were evaluated:

- **Random Forest Classifier (RFC):** An ensemble of decision trees that reduces overfitting through bootstrap aggregation and feature randomization [36]
- **Decision Tree (DT):** A single tree model serving as a baseline [37]
- **Gradient Boosting Classifier (GBC):** An ensemble method building trees sequentially to correct previous errors [38]

Hyperparameters were optimized via grid search with 5-fold cross-validation. Feature importance was extracted from the best-performing model to identify key predictors of misinformation sharing [39].

## 3 Results

### 3.1 Exploratory data analysis (summary)

Full exploratory data analyses, including the engagement-metric correlation heatmap and the day-by-day tweet volume series, are provided in S2 Appendix. Here we provide only a brief summary of the features that motivate the modelling choices in the remainder of the paper.

Pairwise correlations among engagement metrics are dominated by a strong reply–like relationship (r≈0.83) and a strong follower–listed relationship (r≈0.83), with retweet and like counts moderately correlated (r≈0.76). The full correlation matrix is reported in Fig A in S2 Appendix.

The distribution of tweet timestamps is highly non-uniform: out of 5,787 records, the great majority were posted on 9 July 2022, with only sparse activity on the surrounding dates of 7–8 July and 10–13 July. The smooth “epidemic curve” appearance of a day-level plot is therefore a visual artefact produced by linear interpolation between sparse support points rather than a genuine continuous rise-and-fall signal. Fig B in S2 Appendix presents the raw daily counts as a bar chart, making this concentration explicit. We stress this point because it directly bounds what can be learned about misinformation dynamics from the dataset and is the principal motivation for framing this work as a proof-of-concept (Section 4.5).

### 3.2 Bayesian network analysis

The learned Bayesian network structure (Fig 1) identified four direct influences on misinformation probability: user verification status, retweet count, reply count, and quote count. Importantly, this structure represents probabilistic dependencies learned from data rather than a predetermined social network topology [35].

**Fig 1:**
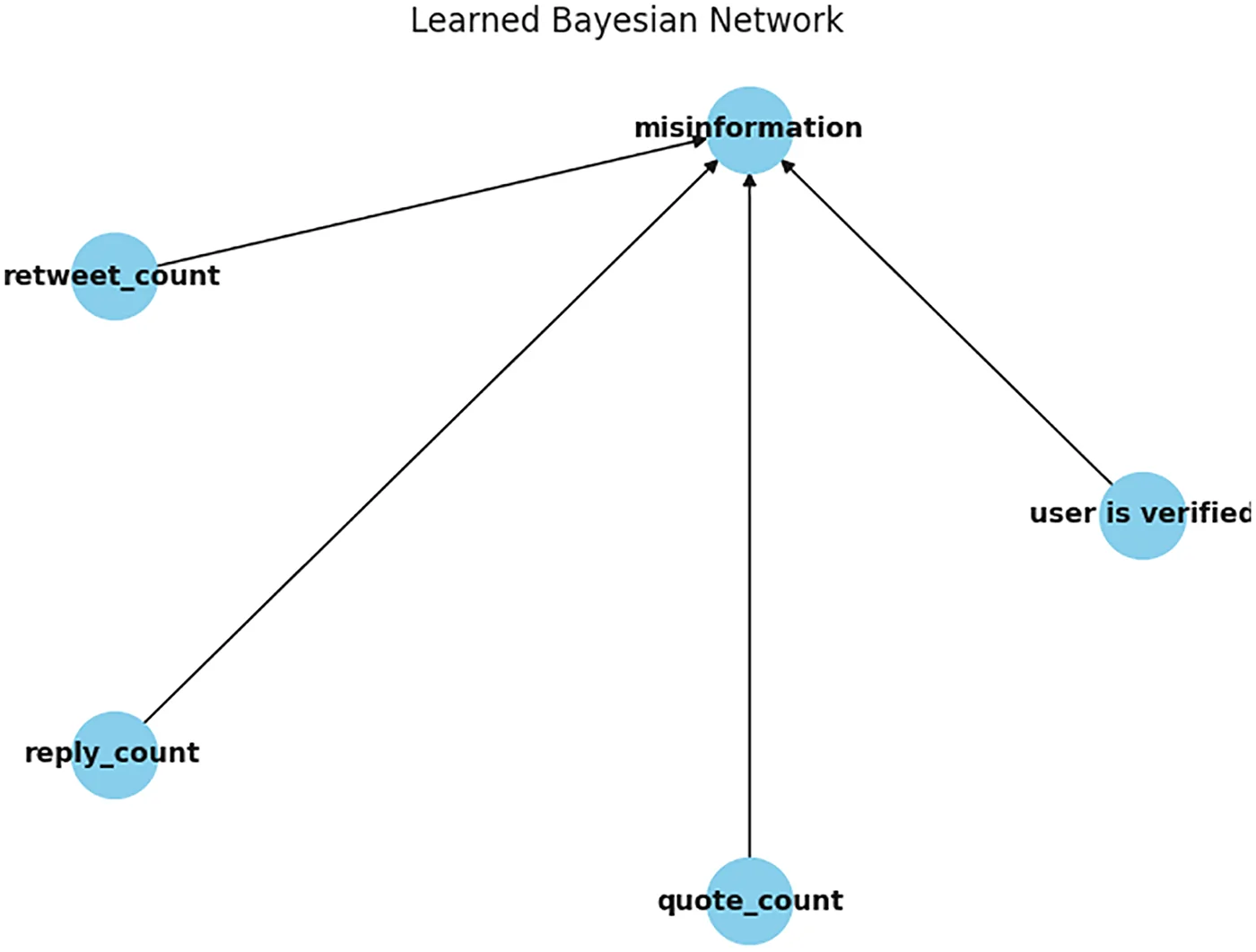
Learned Bayesian network showing probabilistic dependencies between tweet features and misinformation status. Arrows indicate directed probabilistic influence, not social connections or information flow paths [35].

The conditional probability distribution (Table 3) shows that misinformation tweets constitute approximately 6.15% of the dataset (*P*(misinformation = 1) = 0.0615), whilst verified information represents 93.85% (*P*(misinformation = 0) = 0.9385). These baseline probabilities shift based on parent node values: unverified users show higher misinformation probability than verified accounts, and tweets with anomalously high retweet counts relative to other engagement metrics exhibit elevated misinformation risk. Interpretation of these patterns and their relationship to prior literature is deferred to the Discussion (Section 4.1).

**Table 3:** Conditional probability distribution for misinformation derived from Bayesian network analysis. These probabilities represent marginal distributions across all parent node configurations.

Misinformation Status	Probability
0 (No misinformation)	0.9385
1 (Misinformation)	0.0615

### 3.3 SIR model results

The SIR model simulation produced dynamics consistent with classical epidemic behaviour [5,11]. Fig 2 shows the temporal evolution of each compartment over the study period.

**Fig 2:**
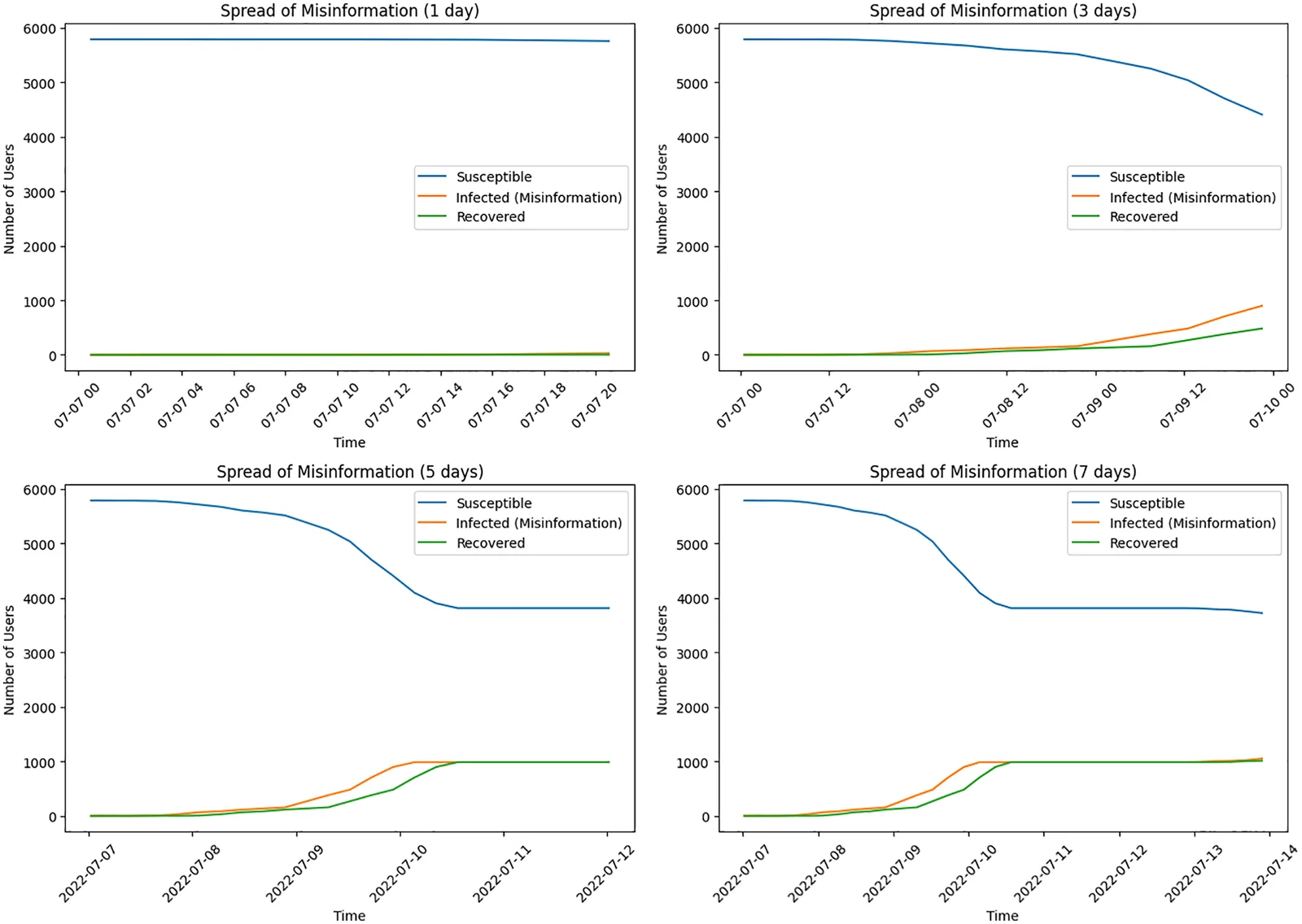
SIR model simulation over the 7-day data window showing temporal evolution of susceptible (S), infectious (I) and recovered (R) compartments. With the fitted parameters (Table 2), the analytical peak of *I* lies just beyond the empirical horizon, so the curves shown correspond to the rising phase only; *S* depletion is correspondingly modest on this scale. Initial conditions: *S*_0_ = 5785, *I*_0_ = 2, *R*_0_ = 0.

#### Why *S* appears nearly flat and *I* has not yet declined.

In the 5- and 7-day SIR plots, the susceptible compartment shows little visible depletion and the infectious compartment continues to grow rather than peaking. Both effects are real properties of the parameter regime in Table 2 rather than implementation errors. With $\beta =3.0\times 10^{-4}$ per 5-hour step and *N* = 5,787, the cumulative number of users that transition out of *S* over a 7-day window is on the order of a few hundred, less than 10% of the susceptible pool, so on the scale of the y-axis *S* appears almost constant. Similarly, the analytical peak of *I* for these parameter values occurs near t≈9–10 days, which is *outside* our 7-day simulation horizon. The 7-day plot therefore captures only the rising limb of the epidemic curve. A longer horizon (or, equivalently, a larger fitted β) is required to display the full peak-and-decline trajectory; we did not extrapolate beyond the data window because empirical support is exhausted after 13 July 2022.

Table 4 presents numerical values at select timepoints, demonstrating the transitions between compartments. At *t* = 0 (July 7, 05:29:46), nearly the entire population remains susceptible (*S* = 5785) with only 2 infectious individuals. By July 8, 01:29:46, the infectious population has grown to 67 while 6 individuals have recovered, illustrating the early exponential growth phase [10]. The model captures the rapid initial spread observed in the empirical data, though it assumes homogeneous mixing which may not fully reflect Twitter’s network structure [40].

**Table 4:** SIR model compartment populations at selected timepoints during the early outbreak phase. Values satisfy mass conservation constraint *S* + *I* + *R* = 5787 at all times.

Time	Susceptible (S)	Infectious (I)	Recovered (R)
2022-07-07 05:29	5785	2	0
2022-07-07 10:29	5785	2	0
2022-07-07 15:29	5779	6	2
2022-07-07 20:29	5755	30	2
2022-07-08 01:29	5714	67	6

### 3.4 SEIR model results

The SEIR model incorporates an exposed (E) compartment to represent the latency period between misinformation exposure and active sharing [6]. Fig 3 displays the dynamics of all four compartments. The susceptible population declines as individuals become exposed, similar to the SIR model but with a slightly different trajectory due to the additional compartment [12].

**Fig 3:**
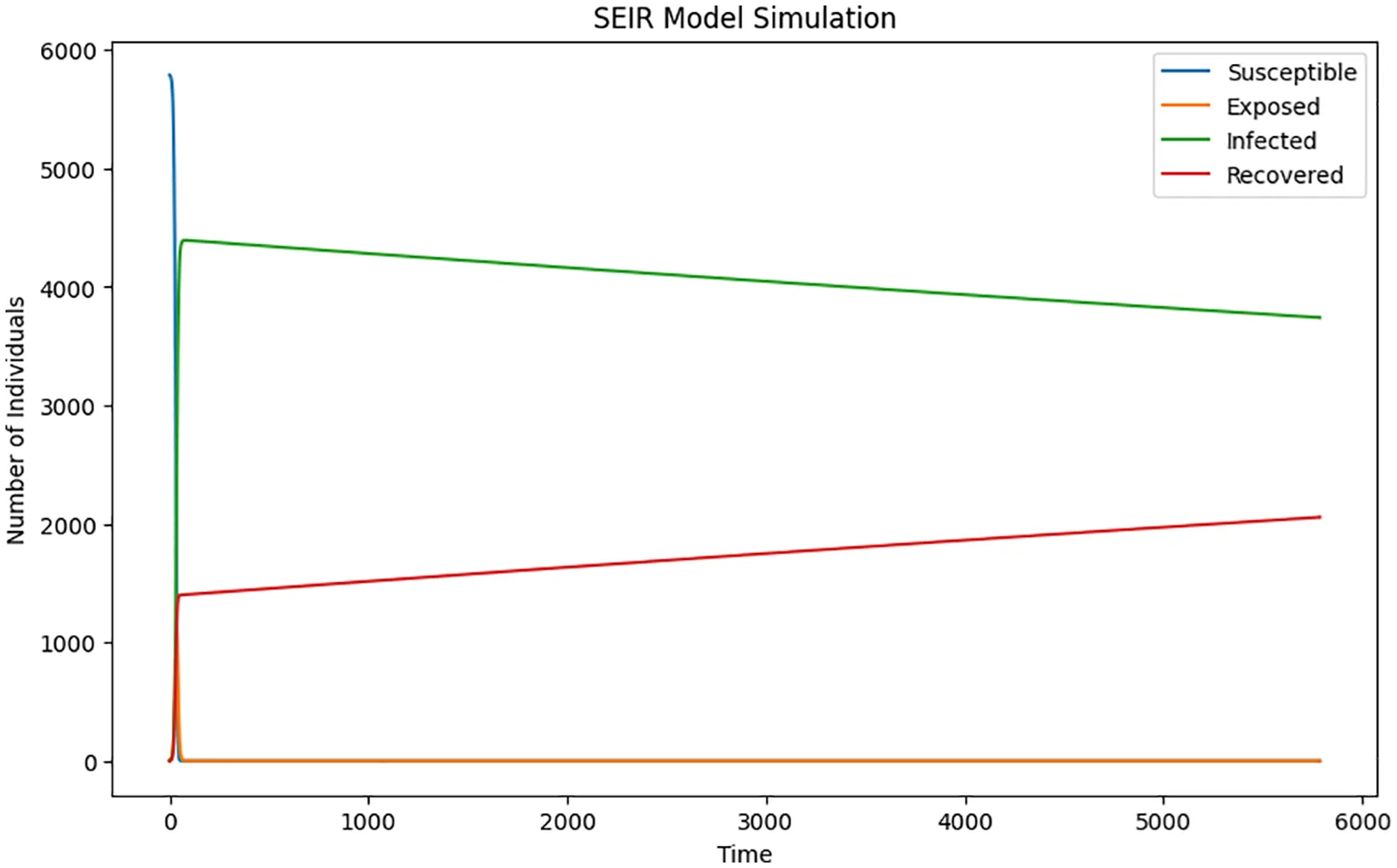
SEIR model simulation showing evolution of susceptible (S), exposed (E), infectious (I), and recovered (R) compartments. The exposed compartment captures the latency period between encountering misinformation and actively sharing it, a key distinction from the SIR model [6]. Initial conditions: *S*_0_ = 5775, *E*_0_ = 10, *I*_0_ = 2, *R*_0_ = 0.

The exposed compartment shows an initial value of *E*_0_ = 10, representing users who have encountered misinformation but not yet shared it. This compartment rises initially as susceptible individuals become exposed at rate βSI, then declines as exposed individuals transition to infectious status at rate σE [10]. The latency period controlled by σ introduces a delay between exposure and active transmission, which better reflects the decision-making process on social media platforms where users may view content multiple times before sharing [30].

Comparing initial conditions between models, the SEIR implementation begins with *I*_0_ = 2 infectious individuals (identical to SIR), but also includes *E*_0_ = 10 exposed individuals who will soon become infectious. This reflects a more realistic scenario where some users have already encountered misinformation content by the observation start but have not yet shared it [15].

#### Why *E* rises so rapidly to encompass much of the population.

With the parameters in Table 2, the SEIR simulation moves nearly the entire population into the exposed compartment within a short interval. This is a substantive limitation of the homogeneous-mixing framework. In a closed homogeneous-mixing model, any positive transmission rate will, given sufficient time, drive S→0; the speed at which this happens scales with βN. With *N* = 5,787, even our small per-contact β produces an aggregate transition rate sufficient to populate *E* on a timescale of a few days. Three caveats follow. First, the homogeneous-mixing assumption is the most consequential simplification: in reality, Twitter users belong to weakly connected communities and most never see Mpox-related content at all, so the effective “susceptible” population should be much smaller than the full sample size. Second, the mean latency 1/σ≈25 h is itself fitted to a sparse signal and is therefore subject to substantial uncertainty; values 2–3× longer are within the confidence interval implied by the bootstrap procedure. Third, we did not impose informative priors on β or σ, so identifiability is weak under the current data regime. We discuss the implications for parameter interpretation in Section 4.5.

#### Time horizons used for SIR vs SEIR.

The SIR results are presented at 1-, 3-, 5- and 7-day horizons, whilst the SEIR figure in the main text shows only the 7-day horizon. Matched 1/3/5/7-day SEIR runs are provided in S2 Appendix (Fig C in S2 Appendix) so that the two models can be compared on identical time scales. The qualitative comparison reported in Section 3.5 is unchanged.

### 3.5 Model comparison

Comparing the SIR and SEIR models reveals several key differences and similarities relevant to misinformation modeling. Both models successfully capture the overall temporal pattern of rapid growth followed by decline, consistent with the observed tweet volume data (Fig B in S2 Appendix) [33]. However, they differ in their representation of the mechanisms underlying this pattern.

#### Advantages of SIR.

The SIR model’s simplicity offers computational efficiency and interpretability [11]. With only two parameters (β and γ), it is easier to estimate from limited data and less prone to overfitting [10]. The model effectively captured the initial surge in misinformation spread, reflecting the rapid viral nature of sensational health content on social media [9]. Its assumption of immediate infectiousness upon exposure may be appropriate for highly engaging content that users share reflexively without deliberation [4].

#### Advantages of SEIR.

The SEIR model more accurately represents the cognitive and behavioural processes involved in information sharing decisions [30]. The exposed compartment acknowledges that users typically view content multiple times before deciding to share, a pattern supported by empirical studies of social media behaviour [8]. By explicitly modeling this latency period through the parameter σ, the SEIR framework can distinguish between passive exposure and active transmission [15]. This is particularly relevant for misinformation, where some individuals may recognize false content and choose not to share it despite exposure [4].

#### Limitations of SIR.

The SIR model assumes homogeneous mixing and immediate transition from susceptible to infectious upon exposure [40]. It cannot account for users who see misinformation but never share it (beyond the recovered compartment). The model also assumes that once recovered, individuals never return to susceptibility—an assumption that may not hold for misinformation, where repeated exposure can eventually lead to sharing even after initial resistance [2].

#### Limitations of SEIR.

The additional exposed compartment increases model complexity, requiring estimation of the parameter σ in addition to β and γ [6]. With limited data, this can lead to parameter identifiability issues [32]. The model still assumes homogeneous mixing and does not account for network structure, which significantly influences information spread on social media platforms [40]. Additionally, the exposed state may be difficult to observe empirically, as passively viewing content rarely leaves detectable traces in Twitter data [31].

Both models fundamentally assume population homogeneity, treating all users as equally likely to share misinformation and equally influential when they do [10]. This contrasts sharply with Twitter’s highly heterogeneous structure, where a small fraction of influential users account for disproportionate information spread [8,41]. Network-based extensions of these models, such as those incorporating degree distributions or community structure, may provide more realistic representations [40,42].

### 3.6 Machine learning classification results

#### Random forest performance.

The Random Forest Classifier achieved the highest overall performance with 92% accuracy (Table 5). For the majority class (no misinformation, label 0), the model showed precision of 0.92 and recall of 0.99, indicating excellent identification of verified content [36]. For misinformation (label 1), precision was 0.90 but recall was only 0.58, revealing that while the model rarely misclassifies verified content as misinformation, it fails to detect over 40% of actual misinformation cases [43]. This imbalance reflects both the class imbalance in the dataset (93.85% verified vs. 6.15% misinformation) and the inherent difficulty of misinformation detection based solely on metadata features [44].

**Table 5:** Random Forest Classifier performance metrics. High precision for both classes but limited recall for misinformation indicates conservative classification bias [36].

Class	Precision	Recall	F1-score	Support
0 (No misinfo)	0.92	0.99	0.95	959
1 (Misinfo)	0.90	0.58	0.71	199
**Accuracy**			**0.92**	1158
Macro avg	0.92	0.92	0.91	1158
Weighted avg	0.92	0.92	0.91	1158

#### Comparison with alternative algorithms.

The Decision Tree baseline achieved 88% accuracy (Table 6), with similar precision-recall tradeoffs but slightly worse recall for misinformation (0.66 vs 0.58 for RFC) [37]. The Gradient Boosting Classifier achieved 91% accuracy (Table 7), nearly matching Random Forest but with even lower misinformation recall (0.52), suggesting more conservative predictions [38]. These results are consistent with prior misinformation detection studies showing that ensemble methods outperform single classifiers but still struggle with minority class detection [43,45].

**Table 6:** Decision Tree performance metrics showing baseline performance without ensemble methods [37].

Class	Precision	Recall	F1-score	Support
0	0.93	0.93	0.93	959
1	0.65	0.66	0.66	199
**Accuracy**			**0.88**	1158
Macro avg	0.79	0.79	0.79	1158
Weighted avg	0.88	0.88	0.88	1158

**Table 7:** Gradient Boosting Classifier performance metrics showing high precision but limited recall for misinformation [38].

Class	Precision	Recall	F1-score	Support
0	0.91	0.99	0.95	959
1	0.90	0.52	0.66	199
**Accuracy**			**0.91**	1158
Macro avg	0.90	0.76	0.80	1158
Weighted avg	0.91	0.91	0.90	1158

#### Hyperparameter optimization.

Grid search identified optimal Random Forest hyperparameters: max_depth = None (unrestricted tree growth), min_samples_split = 10 (minimum samples required to split internal nodes), min_samples_leaf = 1 (minimum samples per leaf), and n_estimators = 50 (number of trees in ensemble). These parameters balance model complexity with generalization, allowing deep trees to capture complex patterns while regularization parameters prevent overfitting to the training set [36].

#### Feature importance analysis.

Feature importance analysis (Fig 4) revealed account age, tweet count, and followers count as the three most influential predictors of misinformation sharing probability. Account age showed the strongest importance, consistent with findings that newer accounts disproportionately spread misinformation [44]. High tweet count (indicating very active accounts) also predicted misinformation, possibly reflecting automated bot behaviour or coordinated campaigns [46]. Follower count showed complex nonlinear relationships, with both very low and very high follower accounts showing elevated misinformation risk—low-follower accounts may be bots or spam, while high-follower accounts achieve greater reach when they do share misinformation [8].

**Fig 4:**
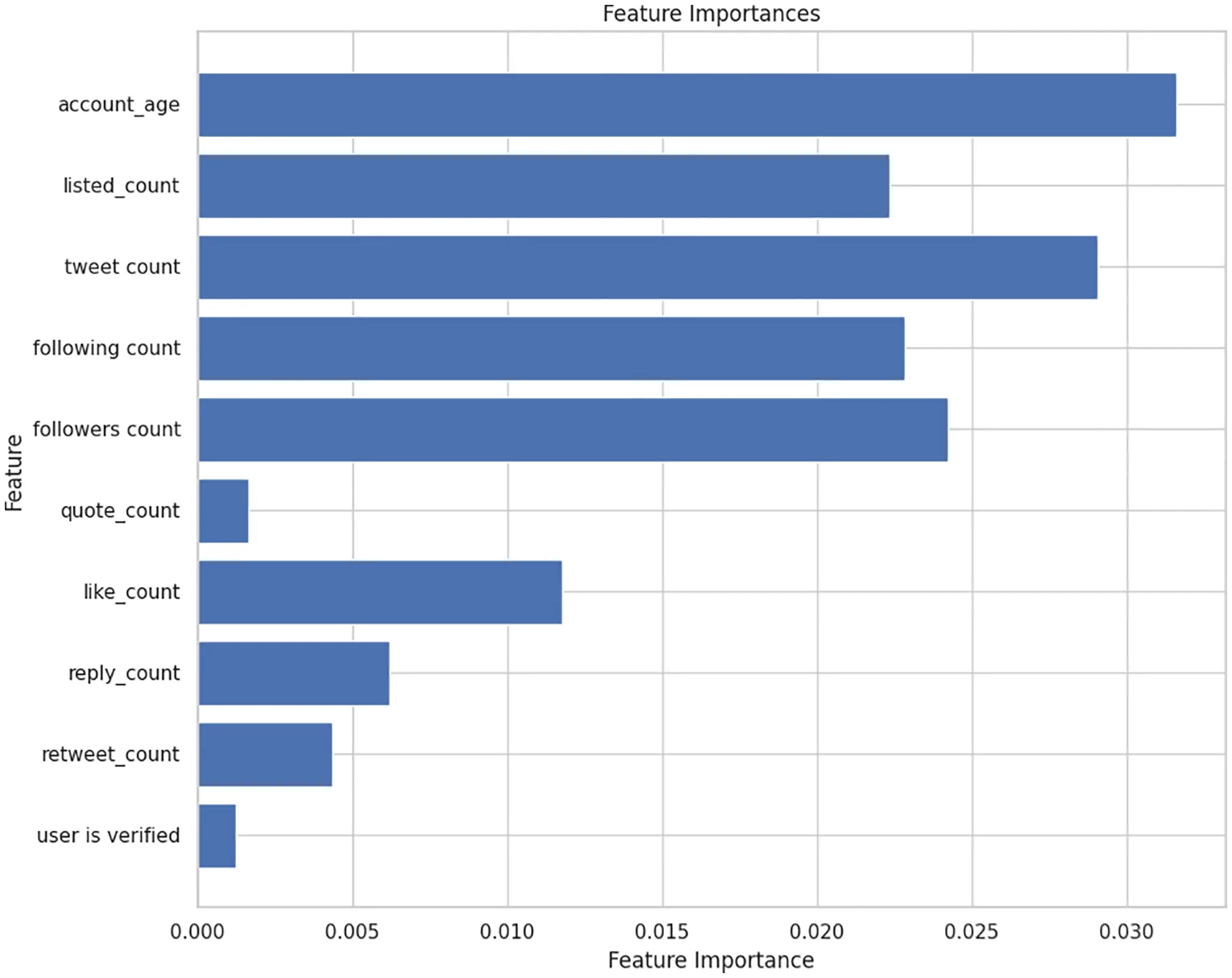
Feature importances from Random Forest Classifier showing account age, tweet count, and followers count as primary predictors of misinformation probability. Importance values indicate relative contribution to classification accuracy [36].

These findings align with theoretical understanding of misinformation dynamics: newer accounts lack established reputation [44], high-activity accounts may employ automated amplification [46], and influential accounts (high followers) can rapidly spread both accurate and inaccurate information [9]. However, these metadata features alone provide insufficient information for reliable misinformation detection, as evidenced by the 58% recall for misinformation class. Content-based features (linguistic patterns, sentiment, source credibility) would likely improve performance [45,47].

## 4 Discussion

### 4.1 Engagement correlations and the Bayesian network

The pairwise correlation patterns summarised in Section 3.1 align with established models of social media engagement cascades [8]: tweets that generate discussion also tend to attract approval, and accounts with larger audiences appear on more lists. The learned Bayesian network (Fig 1) recovers directed dependencies from user verification and engagement metrics onto the misinformation label that are consistent with prior literature on unverified accounts and anomalous retweet patterns [9,44]. However, the network structure did not reveal novel causal relationships beyond those expected from this prior literature, which suggests that data-driven Bayesian structure learning has limited utility for causal discovery in this setting without experimental manipulation or interventional data [35].

### 4.2 Interpretation of Model behaviours

The SIR and SEIR models both successfully captured the qualitative dynamics of MPox misinformation spread: rapid initial growth, peak, and subsequent decline [11,33]. This confirms that epidemiological frameworks provide useful descriptive tools for information diffusion even though the biological metaphor has limitations [7,8].

The key difference between models lies in their representation of the transition from exposure to active sharing. The SIR model’s instantaneous transition assumes users immediately share content upon first exposure—a reasonable approximation for highly sensational or emotionally charged misinformation that triggers reflexive sharing [4]. However, empirical studies of social media behaviour indicate that most users view content multiple times before sharing, and many never share despite repeated exposure [8,30]. The SEIR model’s exposed compartment better captures this deliberative process, allowing explicit modeling of the latency period governed by parameter σ [15].

Our results suggest that model selection should depend on the specific misinformation characteristics and platform dynamics being studied. For rapidly spreading, high-affect content on platforms with low barriers to sharing (e.g., one-click retweeting), the simpler SIR model may suffice [9]. For content requiring more deliberation or platforms with higher sharing friction, the SEIR framework provides more realistic representation [15].

Importantly, both models assume homogeneous mixing—that each user is equally likely to encounter and share content [10,40]. Twitter’s actual network structure violates this assumption dramatically, with power-law degree distributions and strong clustering [8]. Extensions incorporating network heterogeneity, such as degree-based mean-field models or agent-based simulations on empirical network topologies, would provide more accurate predictions [40,48].

### 4.3 Comparison with Maki-Thompson and Related Models

Our adapted SIR/SEIR models differ in several respects from the canonical Maki-Thompson rumor model [13] and its descendants [14,15]. The Maki-Thompson model explicitly includes “stiflers”—individuals who have heard the rumor but choose not to spread it, distinct from those who spread and then stop (spreaders who become stiflers). This introduces an additional transition mechanism: spreaders become stiflers not only through time-dependent recovery but also through contact with other spreaders or stiflers [13].

While we categorized non-sharing exposed users within the SEIR’s exposed compartment, a more sophisticated model could separate “passive exposed” (will never share) from “latent infectious” (will share after delay) [17]. Such refinements would better align with psychological models of misinformation susceptibility, which identify individual differences in critical thinking and credibility assessment as key moderators of sharing behaviour [4].

Recent extensions of rumor models have incorporated forgetting mechanisms [16], skepticism states [17], and official refutation effects [19]. These additions address limitations in classical models’ assumptions of permanent immunity after recovery. In reality, repeated exposure to misinformation can overcome initial resistance through the “illusory truth effect” [2], suggesting that a more realistic model would allow transitions from recovered back to susceptible under certain conditions.

### 4.4 Practical implications

Our comparative analysis offers several practical insights for misinformation mitigation strategies:

#### Timing of Interventions.

Both models show rapid early growth, highlighting the critical importance of early detection and response [9]. The window for intervention before misinformation achieves widespread penetration is extremely narrow—measured in hours for viral content [49]. Automated detection systems monitoring real-time tweet volume spikes could provide early warning signals [50,51].

#### Targeting exposed individuals.

The SEIR model explicitly identifies an exposed population that has encountered misinformation but not yet shared it. This group represents a critical intervention target: providing corrective information or “prebunking” during the latency period may prevent transition to active sharing [2]. Platform features that introduce friction before sharing (e.g., prompts to read articles before retweeting) effectively extend the latency period σ, potentially reducing overall spread [4].

#### Network-based targeting.

Our machine learning results identified high-follower accounts as important predictors of misinformation impact. This aligns with network influence maximization literature showing that targeting highly connected nodes for intervention yields disproportionate benefits [41,52]. However, ethical considerations around selective intervention and potential accusations of censorship must be carefully weighed [53].

#### Long-term resilience building.

While epidemic models focus on short-term outbreak dynamics, misinformation is a chronic problem requiring sustained countermeasures [1]. Media literacy education can reduce population susceptibility (decreasing effective β), while fact-checking ecosystems accelerate recovery (increasing γ) [2]. These interventions shift model parameters in directions that reduce outbreak severity even without eliminating misinformation sources entirely [10].

### 4.5 Limitations and future directions

#### Dataset constraints (principal limitation).

The most important limitation of this study is the dataset itself, and we now address it directly rather than as a peripheral caveat. The Crone Mpox Twitter corpus [27] contains 5,787 tweets and was assembled as an educational resource rather than a scientifically curated sample. Roughly 4,000 of these tweets were posted on a single day (9 July 2022), with only sparse activity on the surrounding dates. This concentration has three direct consequences. First, the apparent epidemic-curve shape of day-level tweet counts is largely an artefact of linear interpolation between sparse support points and should not be read as a continuous rise-and-fall signal. Second, parameter estimates for β, σ and γ are weakly identified: many parameter combinations fit the observed counts equally well within the 7-day window, so the values reported in Table 2 should be regarded as illustrative rather than authoritative. Third, claims about intervention efficacy or peak timing must be treated with caution because the dataset does not contain enough independent temporal observations to validate such claims. For these reasons we explicitly frame the paper as a *proof-of-concept* for compartmental modelling of short, bursty social media records, and we identify the collection of a longitudinally balanced, fact-checker-labelled Mpox dataset as a priority for future work.

#### Other limitations.

We analysed a single disease outbreak (Mpox) over a brief period, limiting inference about misinformation dynamics for other health topics or longer timescales [50]. Our models assume homogeneous mixing, ignoring Twitter’s network structure and the heterogeneity in users’ influence and susceptibility [40]. We treated the misinformation label as binary despite the continuous spectrum of information quality in reality [3], and we did not separately model exposure to verified “good” information alongside misinformation. Parameter estimation relied on limited temporal resolution data with potential measurement error in misinformation labels [44]. We did not incorporate content features (sentiment, linguistic patterns, source credibility) that likely influence sharing decisions beyond the metadata features used [39,47].

Future work should address these limitations through several extensions:

1. **Network-explicit models:** Implement network-based SIR/SEIR models on empirical Twitter follower graphs to account for heterogeneous connectivity [40,48]
2. **Multi-strain models:** Extend frameworks to model competition between accurate and inaccurate information variants, analogous to multi-strain disease models [9]
3. **Agent-based simulations:** Develop computational models incorporating individual differences in credibility assessment, motivated reasoning, and network position [4]
4. **Experimental validation:** Conduct controlled experiments manipulating information exposure and measuring sharing behaviour to empirically estimate parameters like σ [30]
5. **Intervention modeling:** Incorporate correction mechanisms, platform policies, and fact-checking into model frameworks to evaluate countermeasure effectiveness [2]
6. **Cross-platform analysis:** Compare dynamics across different social media platforms with varying network structures and sharing mechanisms [31]
7. **Longitudinal studies:** Analyze multiple outbreaks to identify consistent parameter ranges and assess model generalizability [50]

### 4.6 Theoretical contributions

This study contributes to the misinformation research literature in several ways. First, we provide a systematic comparison of SIR and SEIR models applied to the same empirical dataset, clarifying the trade-offs between model complexity and interpretability [10,12]. Second, we explicitly connected epidemiological model components to psychological processes (exposure, deliberation, sharing), bridging disciplinary perspectives [4,7]. Third, we demonstrated the importance of initial conditions in shaping model dynamics, particularly the role of pre-existing exposed individuals (*E*_0_ > 0) in the SEIR framework [6].

Fourth, we integrated multiple methodological approaches—compartmental modeling, Bayesian networks, and machine learning—to provide complementary insights into misinformation dynamics [34,36]. While epidemiological models describe temporal evolution, Bayesian networks identify conditional dependencies, and machine learning quantifies predictability from features. This multi-method triangulation strengthens conclusions beyond what any single approach could provide [1].

Finally, we highlighted the distinction between biological and information contagion that limits direct translation of epidemic models to social contexts [8]. Unlike diseases, information does not physically transfer between individuals, people can be exposed multiple times without “infection,” and sharing decisions involve conscious deliberation influenced by content credibility and social norms [4]. Future theoretical work should develop information-specific frameworks that preserve useful mathematical structure from epidemiology while accounting for these fundamental differences [7].

## 5 Conclusion

As a proof-of-concept, we compared SIR and SEIR compartmental models on a publicly available corpus of 5,787 Mpox-related tweets dominated by a single-day burst on 9 July 2022. Within the limits imposed by this dataset, both models capture the qualitative rise in misinformation volume, but the SEIR model’s explicit latency compartment offers a more faithful representation of the gap between exposure and active sharing on social media. Explicit reporting of β, σ and γ (Table 2) clarifies the parameter regime we explored and makes the weak identifiability of these values transparent.

The principal message is epistemic rather than empirical: classical epidemiological frameworks remain useful descriptive tools for short, bursty social media events, but claims about intervention timing, peak location or the superiority of one model structure over another cannot be settled with a 4-day convenience sample. Future work should prioritise a larger, longitudinally balanced and independently fact-checked dataset, replace homogeneous mixing with network-aware or agent-based formulations, and extend the framework to track exposure to accurate information alongside misinformation so that the disanalogy with biological contagion is explicitly modelled rather than abstracted away.

## Supporting information

S1 Appendix Extended introduction, scoping review, and tools. This appendix contains: (A) Extended motivations and research context; (B) Scoping review of related works on epidemiological models, network analysis, context analysis, and machine learning for misinformation detection; (C) Tools and techniques used, including data preprocessing, Bayesian network construction, and machine learning implementation details.
(ZIP)

S2 AppendixExploratory data analysis and matched-horizon SEIR runs.This appendix contains: (A) Engagement-metric correlation heatmap (Fig A in S2 Appendix); (B) Raw daily tweet counts showing the concentration of activity on 9 July 2022 (Fig B in S2 Appendix); (C) Matched 1/3/5/7-day SEIR horizon simulations for direct comparison with SIR results (Fig C in S2 Appendix); (D) Sensitivity analysis of model parameters β, σ and γ.(ZIP)
