# Supplementary material for: Epidemiological models in health misinformation propagation of Mpox conversations
Source: PLOS Digit Health. 2026 Nov 13;5(11):e0001772. doi: 10.1371/journal.pdig.0001772 (PMC13766289; doi:10.1371/journal.pdig.0001772)
Supplement: Supplementary file 1 [file pdig.0001772.s005.pdf]

Response to Reviewers

PDIG-D-24-00603R1 — Epidemiological Models in Health Misinformation Propagation of Mpox Conversations

Udoka Duruaku, Tawakalit Omolabake Agboola, Oluwaseun Ajao

April 2026

Dear Dr. Celi and reviewers,

We thank the editor and both reviewers for the careful and constructive second-round feedback. Below we address each comment in turn. Page and section numbers refer to the revised manuscript. Substantive changes have been made throughout the manuscript and are marked in the tracked-changes version.

Reviewer #1

Reviewer comment: *My biggest concern is that my qualms about the Twitter data remained unaddressed. . . . I still believe the Kaggle dataset to be wholly inappropriate for this analysis due to limitations in its size, scope and timeline. I encouraged the authors to discuss this when introducing the dataset, and framing it as a proof of concept work, or collect their own data set for a more robust analysis.*

Response: We accept this criticism in full and have made it the central framing of the revised manuscript. Specifically:

- The Abstract now explicitly describes the study as a *proof-of-concept* and notes that the dataset is dominated by a single burst day (9 July 2022).
- Section 3.1 ("Data Source, Provenance and Scope") has been rewritten from scratch. It now describes the dataset's provenance (assembled by Stephen Crone as an educational resource and released on Kaggle under open terms), the collection method (keyword-based Twitter API queries), the labelling procedure (manual flagging by the dataset author against contemporaneous WHO/CDC guidance), and the temporal distribution of tweets across 7–13 July 2022.
- A new paragraph explicitly discusses representativeness, stating that the corpus is a convenience sample dominated by one day, and that results should not be read as a general characterisation of Mpox misinformation.
- The Limitations section (Section 5.4) now opens with dataset constraints as the *principal* limitation, discusses weak parameter identifiability under the short horizon, and identifies the collection of a longitudinally balanced, independently fact-checked dataset as the top priority for future work.
- The Conclusion has been rewritten to make the epistemic (as opposed to empirical) nature of the contribution explicit.

Reviewer comment: *My second major comment is that there was no appendix included with this resubmission. ...In my next review, I would like to see this appendix and review it in depth as well.*

Response: We apologise for the oversight at the previous round. The manuscript now includes a full **S0 Appendix** containing: (i) the engagement-metric correlation heatmap moved out of the main text; (ii) the raw daily tweet-count figure, re-plotted to make the single-day dominance visually obvious; (iii) matched 1/3/5/7-day SEIR horizons added in response to Reviewer 3; and (iv) a sensitivity-analysis note. The legacy S1 Appendix (extended introduction and scoping review) is retained for completeness.

Reviewer comment: *Figure 1 is a screenshot of the Kaggle dataset, and is inappropriate for inclusion in this analysis without redoing the table yourself.*

Response: Agreed. The screenshot has been removed. In its place we now provide **Table 1 (tab:dataset_fields)**, a self-constructed table that lists all 16 fields of the dataset together with their data type and role in our modelling. Our table is built from the dataset schema rather than reproducing any content from the source site.

Reviewer comment: *All exploratory data analyses should be simply summarized and moved to an appendix, in particular the correlation figure and temporal figure.*

Response: Done. The correlation heatmap and the daily tweet-volume figure have been removed from the main text and relocated to S0 Appendix (S0 Fig 5 and S0 Fig 6). The main text (Section 4.1) now contains only a short prose summary of the EDA.

Reviewer comment: *I have concerns about Figure 3 in general, how it implies a linear trend over time which is not supported by the data. It does not, in fact, look like an epidemic curve, because there is no data in between July 9th and 13th on which to make that conclusion, and a simple linear interpolation is used instead.*

Response: This is an important point and we have addressed it explicitly. The revised EDA summary now states that the smooth epidemic-curve appearance of the day-level plot is a *linear-interpolation artefact* between sparse support points rather than a continuous rise-and-fall signal. The S0 Appendix version of the figure (S0 Fig 6) is re-plotted as a bar chart at daily resolution so that the concentration on 9 July 2022 is visually unambiguous. The Limitations section now uses this observation as the primary justification for the proof-of-concept framing.

Reviewer #3

Reviewer comment: *In the typical background/methods/results/discussion layout, interpretation of the results in a broader context belongs in the discussion.*

Response: Agreed. We have moved the two interpretive sentences flagged by the reviewer ("These patterns align with established models of social media engagement cascades..." and "the network structure did not reveal novel causal relationships...suggesting that Bayesian networks may have limited utility for causal discovery...") out of the Results section and into a new Discussion subsection (Section 5.1, "Engagement correlations and the Bayesian network"). Other stray interpretive sentences in the Results have been audited and similarly relocated where appropriate.

Reviewer comment: *In the results section, it's not necessary to include citations for statements about the work completed within the paper.*

Response: We have removed the unnecessary citations from the Bayesian results paragraph (notably the references to Shu et al. 2017 and Vosoughi et al. 2018 that followed statements about our own findings) and audited the rest of the Results section for similar cases.

Reviewer comment: *In the SIR models, it appears in 5 and 7 days that there is no additional depletion of susceptible although there are infectious people in the population; can you comment on this further? . . . there is no visible decline in the number of infected in any of the SIR visuals.*

Response: We thank the reviewer for catching the inconsistency between the previous draft's text and the figures. The figures are correct; the previous wording was not. With $\beta = 3.0 \times 10^{-4}$ per 5-hour step and $N = 5{,}787$, the cumulative transition out of S over 7 days is on the order of a few hundred, which is visually negligible on the scale of the y-axis; and the analytical peak of I for these parameters occurs at roughly $t \approx 9$–10 days, *beyond* the 7-day empirical horizon. The plots therefore show only the rising limb of the curve. We have: (i) added a new paragraph in Section 4.3 explaining this directly; (ii) updated the figure caption to reflect the rising-phase interpretation; and (iii) removed the previous text that claimed the peak-and-decline was visible in the figure.

Reviewer comment: *I was unclear why the authors didn't state the specific model parameters used (specifically the β, γ and σ); please see an example of how parameters were laid out in a recent modelling paper.*

Response: We have added **Table 1 (tab:model_params)** in Section 3.3.3, which reports the specific values of β, σ, γ, Δt, initial conditions, simulation horizons, and the effective $R_0 \approx 1.7$ for both models. The layout follows the example paper the reviewer provided. We also explain that the mean time in compartment I (i.e. $1/\gamma$) is on the order of tens of hours and should be interpreted as the timescale over which a user remains actively engaged with a given piece of content, not as a biological recovery period.

Reviewer comment: *In particular, the recovery rate is critical to know as the time scale studied is very short . . . I see the authors mention 5 hours, but wasn't clear if that was for the recovery rate or the timing for someone to be still considered infectious.*

Response: Clarified in Section 3.3.3: the *5 hours* is the discrete time step Δt used in the numerical integration, not the recovery time. The mean time an individual spends in the infectious compartment is $1/\gamma \approx 50$ h and the mean latency in the SEIR model is $1/\sigma \approx 25$ h. These values are now explicitly tabulated.

Reviewer comment: *The above point on parameters is especially important for the SEIR model – it appears as though almost the entire population is immediately exposed; could you comment more on this assumption, and uncertainty there?*

Response: Addressed in a new paragraph at the end of Section 4.4 titled "Why E rises so rapidly to encompass much of the population." We explain that this behaviour is a direct consequence of the homogeneous-mixing assumption combined with the scale of βN: in a closed compartmental model any positive transmission rate eventually drives $S \to 0$, and the timescale for this scales with βN. We flag three caveats: (i) homogeneous mixing is unrealistic for Twitter, and the effective susceptible population should be much smaller; (ii) $1/\sigma$ is itself only weakly identified under the current data window and values 2–3$\times$ longer lie within the bootstrap

confidence interval; and (iii) no informative priors were used. The implications are revisited in the Limitations section.

Reviewer comment: *The dataset used includes misinformation/other categories and misinformation/good information categorizations. Why did the authors chose misinformation/other vs misinformation/good?*

Response: We have added a paragraph to Section 3.2 ("Label coding: misinformation vs. other") addressing this directly. We chose the misinformation/other dichotomy because (i) the source dataset does not cleanly separate a "good information" label from "other"; and (ii) compartmental models treat exposure as exposure to the modelled pathogen, so the natural dichotomy is "encountered/shared misinformation or not." We explicitly acknowledge that this departs from the lived experience of social media users (who encounter both accurate and inaccurate content simultaneously), which is an important disanalogy with biological contagion, and we flag competing-strain or two-pathogen extensions as important future work.

Reviewer comment: *Was there a specific reason why the authors didn't do a 1/3/5/7 day analysis for the SEIR model compared to the SIR model, or why there have different time scales?*

Response: No principled reason. We have now run the SEIR model at matched 1/3/5/7-day horizons and report the results in S0 Appendix. The qualitative comparison in Section 4.5 is unchanged, and the main text now explicitly notes that the horizons are matched.

Reviewer comment: *While I appreciate the authors' attention to detail, the length of the conclusion relative to the content of the paper is a bit long; given that the conclusions are mostly stated in the discussion section, I would recommend this be shortened to one or two paragraphs maximum.*

Response: The Conclusion has been cut to exactly two paragraphs. The first summarises the proof-of-concept framing and the SIR/SEIR contrast under the reported parameters; the second states the epistemic message and identifies the top priorities for future work. Points that previously appeared in both the Discussion and the Conclusion have been retained only in the Discussion.

We hope that these revisions satisfactorily address the remaining concerns. We are grateful to both reviewers for the time and care they have invested in improving the manuscript.

Yours sincerely,
Udoka Duruaku, Tawakalit Omolabake Agboola, and Oluwaseun Ajao
corresponding author: `s.ajao@mmu.ac.uk`
