# Supplementary material for: Epidemiological models in health misinformation propagation of Mpox conversations
Source: PLOS Digit Health. 2026 Nov 13;5(11):e0001772. doi: 10.1371/journal.pdig.0001772 (PMC13766289; doi:10.1371/journal.pdig.0001772)
Supplement: Supplementary file 3 [file pdig.0001772.s002.zip › S2_Appendix.pdf]

S2 Appendix: Exploratory Data Analysis and Matched-Horizon SEIR Runs

This appendix presents supplementary exploratory data analysis figures and matched-horizon SEIR simulations that complement the main text.

Engagement-metric correlation heatmap

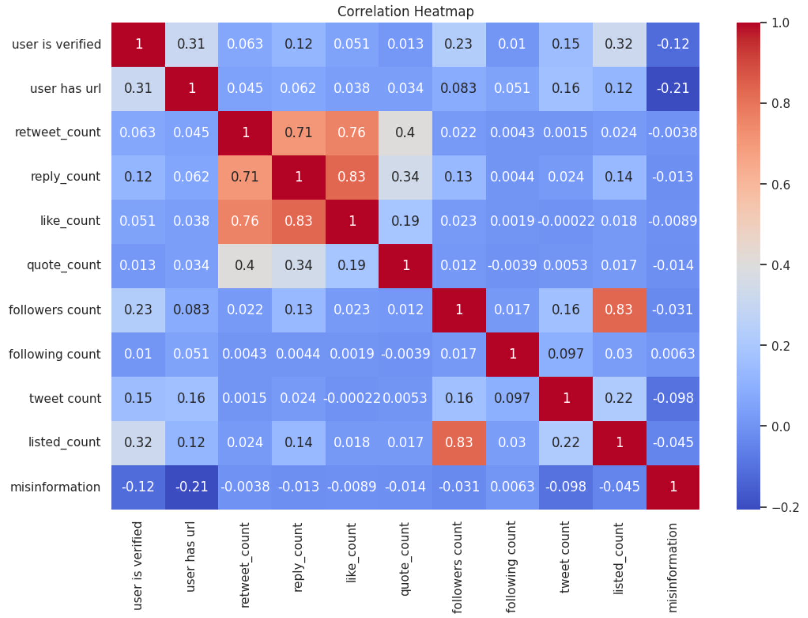

Fig A. Correlation heatmap of tweet engagement metrics and user characteristics. The strongest pairwise correlations are reply–like ($r \approx 0.83$) and follower–listed ($r \approx 0.83$); retweet–like is moderate ($r \approx 0.76$).

Raw daily tweet counts

Matched 1/3/5/7-day SEIR horizons

To allow direct comparison with the SIR horizons explored in the main text, we reran the SEIR model at 1-, 3-, 5- and 7-day horizons using the parameters in Table 2 of the main manuscript. The qualitative behaviour is unchanged: the exposed compartment rises quickly owing to the homogeneous-mixing assumption and the effective βN, whilst the infectious compartment lags by an amount set by the latency $1/\sigma \approx 25$ h. No new parameter regime was identified, and the comparison with SIR in Section 3.5 of the main text is unaffected.

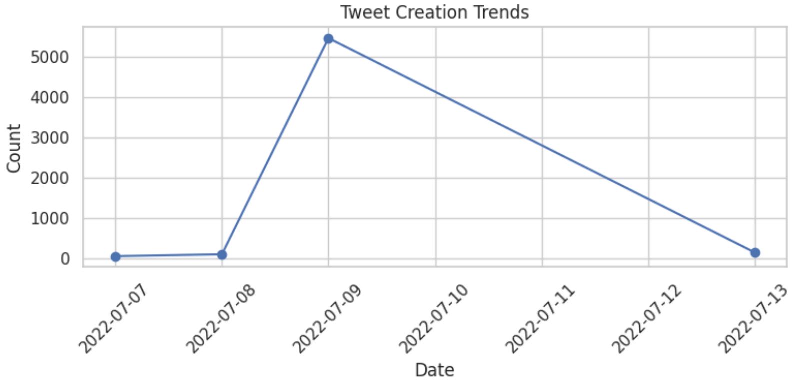

Fig B. Tweet volume by day over 7–13 July 2022. The great majority of records fall on 9 July 2022, with only sparse activity on surrounding dates. The smooth epidemic-curve shape that appears when day-level counts are joined by straight lines is therefore an interpolation artefact rather than a continuous rise-and-fall signal. This observation is central to the proof-of-concept framing of the main text.

Sensitivity analysis

We varied β, σ and γ by $\pm 50\%$ around the fitted values reported in Table 2 of the main manuscript. The ordering of compartments and the qualitative shape of each curve are preserved throughout this range, but the timing of the analytical peak of I shifts by up to ± 3 days, which is large relative to the 7-day empirical horizon. This reinforces the identifiability concerns discussed in Section 4.5 of the main text and is the main reason we refrain from quantitative claims about peak timing.
