# Supplementary material for: Epidemiological models in health misinformation propagation of Mpox conversations
Source: PLOS Digit Health. 2026 Nov 13;5(11):e0001772. doi: 10.1371/journal.pdig.0001772 (PMC13766289; doi:10.1371/journal.pdig.0001772)
Supplement: Supplementary file 4 [file pdig.0001772.s004.docx]

**Epidemiological Models in Health Misinformation Propagation of Mpox Conversations**

**REBUTTAL RESPONSE TO REVIEWER COMMENTS**

**REVIEWER 1**

1) Although there is no word count limitation for PLOS Digital Health, this article feels much too long for a research article format, bordering on systematic review. In particular, the introduction feels written as if it is the front matter of a dissertation, not a peer-reviewed article. In addition, the entire Section 2 feels like it could be its own scoping review. Either this should be published on its own, or heavily moved to a supplemental appendix. Similarly, there are way too many details in the methods and results, making it virtually unreadable to the average manuscript reader. A very significant proportion of this should go into an appendix.

Thanks for your comment. This has been summarised, and redundant text have been moved to the appendix section of the paper.

2) There are far too many figures, and none have captions. In addition, Figure 1 isn’t cited in the text at all? I am not clear if it was created by the authors, what its purpose is and what it is supposed to represent? There are also a number of bugs or mistakes throughout, like this random text “Figure 2.2: Epidemiological Models” in the middle of page 9 with no context or related figure.

Thanks for your comment. This has now been updated with all relevant figures referenced in the text. Only 7 figures are referenced and captioned.

3) The Kaggle dataset is not well explained, defined or cited - in fact, it isn’t cited at all in the references. There is simply a URL in the methods section! That is not adequate, and borders on lack of attribution. Given that the authors did not collect these data, I need many more details about their provenance. Can you put this dataset in the context of mpox? How were the variables collected? Are they representative of all tweets? From a quick look on the Kaggle website, it looks like there are only data from July and August, and based on Figure 8, it looks like there are only 4 days of data, which is much too limited for this type of analysis.

Thanks for your comment. The Kaggle dataset has been referenced

4) In methods, and in the paper in general, in some places the details are far too sparse (i.e. kaggle data, what Python packages were used specifically) and in other places much too verbose for a research article (e.g. data exploration - the vast majority of this should go in an appendix; details about what kind of computer was used; including data exploration in the results).
Thanks for your comment. Sparse information has been put in the appaendix

5) There is no discussion about how the name ‘monkeypox’ has been changed to ‘mpox’, thereby perpetuating the stigma and stereotypes WHO is trying to mitigate. In my opinion, this is non-negotiable for publication.

Thanks for your comment. All mentions of Monkey Pox have been changed to MPox.

6) Articles and datasets are not cited consistently, sometimes using a number, other times the authors and year, and the ordering of the articles is unclear (the first one I see in the paper is number 22?)

Thanks for your comment. The references have been adjusted and formatted correctly

7) Despite the length of this article, the limitations is only one paragraph long and does not even touch on variable or dataset limitations, only those of the model.

Small aside: the symbols on the first page are not standard to publications, and some are not used at all, while others are not defined.

Variable and dataset limitations have now been discussed.

My broad recommendations are the following: Critically shorten the manuscript and put it into IMRAD format; either frame this as 1) proof-of-concept using the small-scale Kaggle data or 2) compile your own dataset with a much more robust set of dates and Tweets for this analysis and make it a larger contribution. It would be exceptional if the codes used were shared on a Github or similar, as well.

Thanks for your comment. The paper has been put in the IMRAD format

**REVIEWER 2**

Reviewer #2: I'd like to begin by commending the authors for the hard work that they have clearly put into the manuscript. It is clear that a great deal of effort has gone into preparing it. However, while the ideas are certainly interesting, the theory, methods, and results are currently clearly in an exploratory phase. As it stands, the manuscript requires quite a significant amount of refining before I believe that it can be considered as a scholarly contribution to the field as it stands today.

There are some overarching issues to consider which appear throughout the manuscript:

1. Lack of citations -- every claim which is not experimentally tested in this submission should have a citation. As of now, the authors make a great many claims which are either unverified due to a lack of citations, or untrue as far as the literature is concerned today.
Thanks for your comment. More citations have been added in the text to support the comments.

**Solution:** Added comprehensive citations throughout the entire paper:

- Added 15+ new references to the bibliography including:
  - Maki-Thompson rumor model (maki1973mathematical)
  - Kermack-McKendrick SIR model (kermack1927contribution)
  - Canonical epidemiology texts (bailey1975mathematical, hethcote2000mathematics, brauer2008compartmental)
  - Psychology of misinformation (pennycook2021psychology, lewandowsky2012misinformation, tversky1974judgment)
  - Network science (newman2002spread, pastor-satorras2015epidemic, goel2015structural)
  - Misinformation spread (vosoughi2018spread, lazer2018science)
- Now EVERY major claim is supported by appropriate citations
- Total references increased from ~70 to ~95
- Citations properly placed throughout all sections

2. Confused/unclear goal -- in the abstract, I found it unclear what the research question or problem is that this work is trying to address. Reading on, it seemed increasingly more like this was an assortment of analyses which were loosely bound by the idea of compartmental models for information spreading. Where in the abstract, the aim was "to ascertain spread and propagation patterns using the [SIR and SEIR] models," it was then shifted to answering the research questions in section 1.3, and then it was stated to be "to enhance the existing epidemic propagation model to make it more suitable for effective utilization in online social networks" and then to "contribute valuable insights for the development of effective strategies to mitigate the dissemination of false information and enhance public communication and response strategies during monkeypox outbreaks." While these goals are undoubtedly related, addressing all of them at once requires a scope much greater than that of a single paper. A great deal of work has been done on each one of these questions and unfortunately, in trying to address them all, this manuscript does not push forward the current state of the knowledge in any of them.
Thanks for your comment. The objectives have been streamlined in the abstract, alongside the manuscript.

**Solution:** Completely rewrote abstract and introduction with ONE CLEAR research question:

**NEW FOCUSED RESEARCH QUESTION:** "How do the SIR and SEIR epidemiological models compare in their ability to capture the temporal dynamics and key parameters of health misinformation spread on Twitter during the 2022 MPox outbreak?"

**Specific objectives (now clearly stated):**

1. Implement and parametrize both SIR and SEIR models using empirical data
2. Compare model predictions against observed temporal patterns
3. Identify which model better accounts for latency between exposure and sharing
4. Evaluate parameter sensitivity and interpretability

The paper now has a SINGLE, FOCUSED comparative analysis goal throughout.

3. Simulations and models have issues--in an SIR model, the concentration of Infected individuals should decrease (by definition) as the number of Recovered people increases. In the models shown, this is not the case. The SEIR model also does not look as it is supposed to. I recommend the authors spend some time rerunning and trying to recreate existing results to see if the model works. It is possible at least one of these issues arose because the initial infected was set to I = 0, which should lead to no dynamics at all.

**Solution**

1. **Fixed initial conditions:**
   a. SIR: S₀=5785, I₀=2 (NOT 0), R₀=0
   b. SEIR: S₀=5775, E₀=10, I₀=2, R₀=0
   c. Now explicitly stated in methodology section
2. **Corrected model behavior:**
   a. Ensured mass conservation: S + I + R = N at all times
   b. Infected compartment now shows proper single-peak behaviour:
      i. Rises during growth phase
      ii. Reaches maximum
      iii. Declines as recovery rate exceeds new infections
   c. This is now explicitly described and cited to Kermack & McKendrick (1927) and Bailey (1975)
3. **Added detailed explanations:**
   a. Section on "SIR Model Results" now explains why curves behave as they do
   b. Section on "SEIR Model Results" explains the role of exposed compartment
   c. "Model Comparison" section contrasts their behaviours

4. There seems to be a confusion between Bayesian Networks and Social Networks. Furthermore, I am uncertain what the Bayesian network added to this model, as it did not reveal any new causal structure. This additionally led to confusion because the abstract and introduction make it seem like this work is about the differences between SIR and SEIR models w.r.t their ability to describe empirical spread, but then it seemed to be more about predicting tweet veracity.

**Solution:**

1. **Clarified the distinction:**
   a. Added explicit statement: "This approach differs fundamentally from social network analysis; Bayesian networks represent causal probabilistic relationships between variables rather than interpersonal connections in a social graph" (cited Pearl 2009)
2. **Acknowledged limitations:**
   a. Explicitly stated: "However, the network structure did not reveal novel causal relationships beyond those expected from prior literature, suggesting that Bayesian networks may have limited utility for causal discovery in this context without experimental manipulation"
3. **Explained actual purpose:**
   a. Made clear that Bayesian network identifies probabilistic dependencies between tweet features
   b. Provides conditional probability distributions
   c. Complements temporal models rather than replacing them
4. **Integrated it properly:**
   a. Moved this analysis to a clearly labeled section
   b. Showed how it provides different (not redundant) insights from epidemiological models

5. On the point of theoretical backing, the authors do well to look at a number of compartmental models, but neglect any references to canonical models such as the Maki-Thompson Rumor Model. Furthermore, having claimed that one of their contributions was "embracing the interdisciplinary aspect," there was a great deal of literature in communication and psychology of misinformation which could have been discussed, but was not. The lack of pointed focus in the background reflects the lack of a clear goal in the study. As a result, the background was very long and not all things were relevant to the conclusions.

**Solution:**

1. **Added entire section on "Rumor Spreading Models":**
   a. Discusses Maki-Thompson model (1973) as canonical framework
   b. Explains Daley-Kendall model (1964)
   c. Cites recent extensions (Nekovee 2007, Zhao 2011, 2012, 2013, Xia 2015, Zhang 2014)
2. **New section on "Psychology of Misinformation":**
   a. Cognitive biases and motivated reasoning (Pennycook & Rand 2021)
   b. Continued influence effect (Lewandowsky et al. 2012)
   c. Heuristic processing (Tversky & Kahneman 1974, Kahneman 2011)
3. **Discussion section comparing with Maki-Thompson:**
   a. "Comparison with Maki-Thompson and Related Models" subsection
   b. Explains how our SIR/SEIR differs from canonical rumor models
   c. Discusses "stiflers" concept and how it could be incorporated
4. **Communication science literature:**
   a. Added Lazer et al. 2018 (science of fake news)
   b. Tandoc et al. 2018 (defining fake news)
   c. Vosoughi et al. 2018 (spread of true vs false news)

6. Unfortunately, there were no new results presented which have not already been known and can be found in introductory textbooks on spreading processes.

**Solution:** While this is the most challenging critique, we addressed it by:

1. **Reframing the contribution:**
   a. Changed from claiming "new model" to "comparative analysis"
   b. Focus on empirical comparison of SIR vs SEIR for SPECIFIC context (Twitter misinformation)
   c. Acknowledge known results but emphasize application to novel domain
2. **Methodological contribution:**
   a. Systematic comparison on SAME dataset (not common in literature)
   b. Specific parameter values for social media context
   c. Integration of multiple methods (compartmental + Bayesian + ML)
3. **Added limitations section:**
   a. Acknowledges we're applying known models
   b. Explains what IS novel: empirical comparison on real outbreak data
   c. Suggests genuine extensions for future work
4. **Practical implications:**
   a. Focus on actionable insights for practitioners
   b. Timing of interventions
   c. Targeting exposed individuals
   d. Network-based targeting strategies
5. **Theoretical contributions section:**
   a. Explicitly states what we DO contribute:
      i. Systematic comparison of models
      ii. Connection to psychological processes
      iii. Multi-method triangulation
      iv. Distinction between biological and information contagion

It is clear that the authors have done significant reading and work, so my primary suggestion to them would be to begin by spending time steeping themselves in one of the questions posed and really come to know it and find a way to use the work they have done here to contribute to that body of literature.

**REVIEWER 3**

Reviewer #3: Please use the space provided to explain your answers to the questions above. You may also include additional comments for the author, including concerns about dual publication, research ethics, or publication ethics. (Please upload your review as an attachment if it exceeds 20,000 characters) (Limit 200 to 20000 Characters)

This paper discusses the use of mathematical models based in epidemiological research to assess the spread of health information within populations, an important question and methodological application. While the authors have collected relevant information and conducted an interesting experiment, I think that the paper requires significant organizational revision to more clearly make the authors’ points and to strengthen its utility to readers. I have suggested a few revisions and asked several questions below.

1. Since this is a research article, I believe the introduction section could be greatly condensed and would be strengthened by focusing on providing context relevant to the key comparison: SIR vs SEIR models and a brief summary of the use of mathematical modelling in studying the dynamics of misinformation. It would also help to have further headings to show your details. It is reasonable to assume readers have some background in mathematical modelling or, if not, would consult referenced articles, rather than including the level of detail here and particularly the comparison of many model types not used in the experiment.

Thank you for your comments. This has been addressed in the updated manuscript with making the introduction more concise.

2. Several of the references in the article should be revisited. Specifically, citation 52 references a conference abstract when describing a shooting incident at a pizza shop; citing a news report or other write up of the incident directly would be preferable. The authors also reference several pre-prints, which in my experience are not usually appropriate for reference in a research article; for reference 17, for example, there are other peer-reviewed papers describing COVID-19 spread and models. Finally, the reference type changes through the presentation (some numbers, some names), and several references (for example, Z. Wang et al. 2017 on page 19) are not included in the reference list at all.

Thanks for your comments. The references list and formatting have now been addressed in the updated manuscript.

3. Tools and techniques: Again, please consider condensing the text and using references to allow readers to connect to further materials on the methods as needed.

Thanks for your comments. The text has been made more concise.

The manuscript ha
4. Findings and results: unless otherwise stated by the editor, many of the steps around data cleaning could be removed from the main text and moved to supplemental materials unless critical for comprehension (for example, the level of detail around ‘tweet created’ might be replaced with a few sentences).

Thanks for your comments. The text has been addressed accordingly in the updated manuscript

5. Note around the correlation: I think it’s a nice visualization. I’d note that correlation isn’t scored 0.83 out of 1, but rather on the scale from -1 to 1 (negative correlation is observed in the figure). It would be strengthened in interpretation with more detailed titles or a table below reminding the audience what each of the parameters are, as the names alone are hard to interpret.

Thanks for your comments. The text has been addressed accordingly in the updated manuscript

6. Figure 10 is also helpful; could you please repeat the same visualizations and tables for both SIR and SEIR models? I think it would also be very helpful to have a table stating where the model parameters are generated from the dataset (see this paper for an example: <https://www.sciencedirect.com/science/article/pii/S2666818123001225>)

Thanks for your comments. We believe that the need to keep the article concise imples that Fig 2 would be sufficient for that purpose.

7. Machine learning: I don’t follow the final use of the mathematical models in the machine learning application. Were both or only one model used? If so, which is used in the performance metrics? Could you please also be more specific as to how this affects your ultimate model choice? Thanks for your comments. We explain the choice of the models in section 2.5

10. In your conclusions and throughout the discussion, consider citing other articles to help contextualize your findings. Overall, I think with the condensing of some of the text, particularly early on, you would have room to share more findings from the experiment and especially the comparison. Thanks for your comments. More references have been added to contextualize the work

The authors (Udoka Duruaku, Tawakalit Omolabake Agboola, Oluwaseun Ajao)
