# Supplementary material for: Epidemiological models in health misinformation propagation of Mpox conversations
Source: PLOS Digit Health. 2026 Nov 13;5(11):e0001772. doi: 10.1371/journal.pdig.0001772 (PMC13766289; doi:10.1371/journal.pdig.0001772)
Supplement: Supplementary file 5 [file pdig.0001772.s006.docx]

**Response to Reviewers**

PLOS Digital Health — Manuscript PDIG-D-24-00603R2

Date: 5 June 2026

**Manuscript title:** Epidemiological Models in Health Misinformation Propagation of Mpox Conversations

**Corresponding author:** Oluwaseun Ajao (s.ajao@mmu.ac.uk)

Dear Editor,

Thank you for the opportunity to submit a final revision, and for Reviewer 1’s constructive feedback. The reviewer’s remaining comments ask us to remove from the manuscript body any passages that read as direct replies to the reviewer, so that the article stands on its own and any explanation of why a change was made is confined to this letter. We agree and have implemented this throughout. Each comment is reproduced below (in italics) with our response. All edits are visible in the marked-up manuscript (“Revised Manuscript with Track Changes”) and incorporated into the clean manuscript.

**Reviewer 1**

**Comment 1 (top of page 5).**

*At the top of page 5, you write “Consistent with reviewer guidance, we describe its provenance, fields and temporal scope in detail and explicitly discuss the implications of its limitations for our findings.” This is quite non-standard for a journal article and I believe should be removed and just explained that the provenance and scope are described in the following section.*

**Response:**

We have removed this sentence. The Data Source, Provenance and Scope subsection now opens directly with the dataset and simply states that its provenance, fields and temporal scope are described in detail in the following subsections, which carry the headings Provenance, Temporal scope, Fields and Representativeness. No reference to the review process remains.

**Comment 2 (page 10).**

*On page 10, there is no need to describe that the “epidemic curve” appearance was an artifact. Removing the mention of the “epidemic curve” appearance altogether is preferable, and keeping your reply to me is adequate.*

**Response:**

We have removed the sentence in the Results (Exploratory Data Analysis summary) that described the “epidemic curve” appearance as a visual artefact. As you suggest, the explanation is retained only here in the response letter and is adequate: because roughly 4,000 of the 5,787 tweets fall on a single day (9 July 2022), a day-level line plot looks like a smooth rise-and-fall only as a consequence of linear interpolation between sparse support points, not because of a genuine continuous signal. The Results text now states the concentration of activity and refers to the raw daily bar chart (S2 Fig 2) without the “epidemic curve” framing.

**Comment 3 (page 21).**

*On page 21, there is no need to mention that you “now” highlight the limitation of the mpox kaggle dataset. Just jump straight into the limitations.*

**Response:**

We have removed the prefatory clause (“…and we now address it directly rather than as a peripheral caveat”). The Limitations and Future Directions section (§4.5) now begins directly with the substantive limitation: “The most important limitation of this study is the dataset itself.”

**General comment.**

*In sum, all of the parts of the manuscript that are pointed replies to the reviewer should be removed. Those belong in the response to reviewers. Making the requested changes and additions is enough for the manuscript itself without being explicit as to why the changes were made.*

**Response:**

We have reviewed the full manuscript and removed every remaining passage framed as a reply to the reviewer or as narration of the revision process, while retaining all of the underlying content and additions. Specifically:

• In the Methods, the clause *“Following reviewer feedback,”* before the description of the model-parameter table has been deleted; the table description itself is unchanged.

• In the Results summary, the phrase *“in response to reviewer guidance”* has been deleted; the statement that the full exploratory analyses were moved to the S2 Appendix remains.

• In the SIR results, the opener *“Reviewer feedback rightly noted that…”* has been removed, and the sentence acknowledging that the previous revision’s wording overstated the visibility of the peak has been deleted. The substantive explanation of why the susceptible compartment appears flat and the infectious compartment has not yet peaked is retained.

• In the model-comparison discussion, the framing *“In response to reviewer feedback we have added…”* has been reworded so that the matched 1/3/5/7-day SEIR runs in the S2 Appendix are simply stated as provided.

We have left the data-sparsity caveat in the Limitations section (§4.5) and the corresponding S2 figure caption, since there it functions as substantive scientific content (motivating the proof-of-concept framing and the weak identifiability of the fitted parameters) rather than as a reply to the reviewer. If you would prefer the “epidemic-curve” wording removed from those two locations as well, we are glad to do so.

We are grateful to Reviewer 1 for feedback that has improved the presentation of the manuscript, and we hope the revised version is now suitable for publication.

Sincerely,

Udoka Duruaku, Tawakalit Omolabake Agboola, and Oluwaseun Ajao

School of Computing and Mathematics, Manchester Metropolitan University
