# Supplementary material for: Epidemiological models in health misinformation propagation of Mpox conversations
Source: PLOS Digit Health. 2026 Nov 13;5(11):e0001772. doi: 10.1371/journal.pdig.0001772 (PMC13766289; doi:10.1371/journal.pdig.0001772)
Supplement: Supplementary file 6 [file pdig.0001772.s007.docx]

Response to Reviewers

PDIG-D-24-00603R2 — Epidemiological Models in Health Misinformation Propagation of Mpox Conversations

Udoka Duruaku, Tawakalit Omolabake Agboola, Oluwaseun Ajao

August 2026

Dear Dr. Celi,

We thank Reviewer 1 for the careful final reading and for confirming that, once the three items below are corrected, all feedback will have been thoroughly incorporated. Each item involved reviewer-facing language that should have been removed before submission; we have now deleted it and verified that no similar traces remain elsewhere in the manuscript.

# Reviewer #1

*Reviewer comment: (1) The caption of Table 2 still says “Reporting follows the layout suggested by the reviewer [33].”*

**Response:** Corrected. The sentence has been deleted from the Table 2 caption. The caption now ends after the statement of the effective basic reproduction number: “...the effective basic reproduction number is *R*_0_ = *βN/γ* ≈ 1*.*7.” The corresponding bibliography entry [33] has also been removed from the reference list.

*Reviewer comment: (2) The caption of Figure 3 retains the following text: “Why E rises so rapidly to encompass much of the population. A reviewer noted that, with the parameters in Table 2, the SEIR simulation moves nearly the entire population into the exposed compartment within a short interval, which appears unrealistic.”*

**Response:** Corrected. The reviewer-facing sentence has been replaced with neutral, manuscriptappropriate language. The passage now reads: “With the parameters in Table 2, the SEIR simulation moves nearly the entire population into the exposed compartment within a short interval. This is a substantive limitation of the homogeneous-mixing framework.” No reference to the review process remains.

*Reviewer comment: (3) Although the language in the main text about appendix S2 has been modified, the S2 appendix itself still has the following language: “S2 Appendix: Exploratory Data Analysis and Matched-Horizon SEIR Runs. This appendix collects the exploratory figures that were previously embedded in the main text, together with the additional 1/3/5/7-day SEIR runs requested by Reviewer 3.”*

**Response:** Corrected. The introductory sentence of S2 Appendix now reads: “This appendix presents supplementary exploratory data analysis figures and matched-horizon SEIR simulations that complement the main text.” All references to the review process and to specific reviewer requests have been removed.

1

**Verification.** We performed a full-text search of the revised manuscript for the terms “reviewer”, “previous revision”, “previous draft”, “resubmission” and “previous round”. No matches were found, confirming that the manuscript is now free of review-process language.

We are grateful for the reviewer’s diligence in catching these oversights and trust that the manuscript is now ready for final consideration.

Yours sincerely,

Udoka Duruaku, Tawakalit Omolabake Agboola, and Oluwaseun Ajao *Corresponding author:* s.ajao@mmu.ac.uk

2
