# Supplementary material for: Epidemiological models in health misinformation propagation of Mpox conversations
Source: PLOS Digit Health. 2026 Nov 13;5(11):e0001772. doi: 10.1371/journal.pdig.0001772 (PMC13766289; doi:10.1371/journal.pdig.0001772)
Supplement: Peer review history

# Peer review: Decision Letter 0

**DOI:** 10.1371/journal.pdig.0001772.r001

**Authors:** J. Ansermino

26 Sep 2025

Response to Reviewers Revised Manuscript with Track Changes Manuscript **Journal Requirements:**

1. Please provide an Author Summary. This should appear in your manuscript between the Abstract (if applicable) and the Introduction, and should be 150–200 words long. The aim should be to make your findings accessible to a wide audience that includes both scientists and non-scientists. Sample summaries can be found on our website under Submission Guidelines: [LINK]

https://journals.plos.org/digitalhealth/s/submission-guidelines#loc-parts-of-a-submission

**Additional Editor Comments (if provided):** **Reviewers' Comments:**

**Comments to the Author**

1. Does this manuscript meet PLOS Digital Health’s publication criteria?

Reviewer #1: Yes

Reviewer #2: No

Reviewer #3: Partly

**********

2. Has the statistical analysis been performed appropriately and rigorously?

Reviewer #1: Yes

Reviewer #2: No

Reviewer #3: Yes

**********

3. Have the authors made all data underlying the findings in their manuscript fully available (please refer to the Data Availability Statement at the start of the manuscript PDF file)?

The PLOS Data policy

Reviewer #1: Yes

Reviewer #2: Yes

Reviewer #3: Yes

**********

4. Is the manuscript presented in an intelligible fashion and written in standard English?

Reviewer #1: No

Reviewer #2: Yes

Reviewer #3: Yes

**********

Reviewer #1: This is a really interesting premise and I thank the authors for their contribution to the study of how misinformation spreads.

That said, I think the current paper needs very extensive revisions to be considered for a publication and before I can even read it through line-by-line.

My broad comments:

1) Although there is no word count limitation for PLOS Digital Health, this article feels much too long for a research article format, bordering on systematic review. In particular, the introduction feels written as if it is the front matter of a dissertation, not a peer-reviewed article. In addition, the entire Section 2 feels like it could be its own scoping review. Either this should be published on its own, or heavily moved to a supplemental appendix. Similarly, there are way too many details in the methods and results, making it virtually unreadable to the average manuscript reader. A very significant proportion of this should go into an appendix.

2) There are far too many figures, and none have captions. In addition, Figure 1 isn’t cited in the text at all? I am not clear if it was created by the authors, what its purpose is and what it is supposed to represent? There are also a number of bugs or mistakes throughout, like this random text “Figure 2.2: Epidemiological Models” in the middle of page 9 with no context or related figure.

3) The Kaggle dataset is not well explained, defined or cited - in fact, it isn’t cited at all in the references. There is simply a URL in the methods section! That is not adequate, and borders on lack of attribution. Given that the authors did not collect these data, I need many more details about their provenance. Can you put this dataset in the context of mpox? How were the variables collected? Are they representative of all tweets? From a quick look on the Kaggle website, it looks like there are only data from July and August, and based on Figure 8, it looks like there are only 4 days of data, which is much too limited for this type of analysis.

4) In methods, and in the paper in general, in some places the details are far too sparse (i.e. kaggle data, what Python packages were used specifically) and in other places much too verbose for a research article (e.g. data exploration - the vast majority of this should go in an appendix; details about what kind of computer was used; including data exploration in the results).

5) There is no discussion about how the name ‘monkeypox’ has been changed to ‘mpox’, thereby perpetuating the stigma and stereotypes WHO is trying to mitigate. In my opinion, this is non-negotiable for publication.

6) Articles and datasets are not cited consistently, sometimes using a number, othertimes the authors and year, and the ordering of the articles is unclear (the first one I see in the paper is number 22?)

7) Despite the length of this article, the limitations is only one paragraph long and does not even touch on variable or dataset limitations, only those of the model.

Small aside: the symbols on the first page are not standard to publications, and some are not used at all, while others are not defined.

My broad recommendations are the following: Critically shorten the manuscript and put it into IMRAD format; either frame this as 1) proof-of-concept using the small-scale Kaggle data or 2) compile your own dataset with a much more robust set of dates and Tweets for this analysis and make it a larger contribution. It would be exceptional if the codes used were shared on a Github or similar, as well.

Reviewer #2: I'd like to begin by commending the authors for the hard work that they have clearly put into the manuscript. It is clear that a great deal of effort has gone into preparing it. However, while the ideas are certainly interesting, the theory, methods, and results are currently clearly in an exploratory phase. As it stands, the manuscript requires quite a significant amount of refining before I believe that it can be considered as a scholarly contribution to the field as it stands today.

There are some overarching issues to consider which appear throughout the manuscript:

1. Lack of citations -- every claim which is not experimentally tested in this submission should have a citation. As of now, the authors make a great many claims which are either unverified due to a lack of citations, or untrue as far as the literature is concerned today.

2. Confused/unclear goal -- in the abstract, I found it unclear what the research question or problem is that this work is trying to address. Reading on, it seemed increasingly more like this was an assortment of analyses which were loosely bound by the idea of compartmental models for information spreading. Where in the abstract, the aim was "to ascertain spread and propagation patterns using the [SIR and SEIR] models," it was then shifted to answering the research questions in section 1.3, and then it was stated to be "to enhance the existing epidemic propagation model to make it more suitable for effective utilization in online social networks" and then to "contribute valuable insights for the development of effective strategies to mitigate the dissemination of false information and enhance publicc communication and response strategies during monkeypox outbreaks." While these goals are undoubtedly related, addressing all of them at once requires a scope much greater than that of a single paper. A great deal of work has been done on each one of these questions and unfortunately, in trying to address them all, this manuscript does not push forward the current state of the knowledge in any of them.

3. Simulations and models have issues--in an SIR model, the concentration of Infected individuals should decrease (by definition) as the number of Recovered people increases. In the models shown, this is not the case. The SEIR model also does not look as it is supposed to. I recommend the authors spend some time rerunning and trying to recreate existing results to see if the model works. It is possible at least one of these issues arose because the initial infected was set to I = 0, which should lead to no dynamics at all.

4. There seems to be a confusion between Bayesian Networks and Social Networks. Furthermore, I am uncertain what the Bayesian network added to this model, as it did not reveal any new causal structure. This additionally led to confusion because the abstract and introduction make it seem like this work is about the differences between SIR and SEIR models w.r.t their ability to describe empirical spread, but then it seemed to be more about predicting tweet veracity.

5. On the point of theoretical backing, the authors do well to look at a number of compartmental models, but neglect any references to canonical models such as the Maki-Thompson Rumor Model. Furthermore, having claimed that one of their contributions was "embracing the interdisciplinary aspect," there was a great deal of literature in communication and psychology of misinformation which could have been discussed, but was not. The lack of pointed focus in the background reflects the lack of a clear goal in the study. As a result, the background was very long and not all things were relevant to the conclusions.

6. Unfortunately, there were no new results presented which have not already been known and can be found in introductory textbooks on spreading processes.

It is clear that the authors have done significant reading and work, so my primary suggestion to them would be to begin by spending time steeping themselves in one of the questions posed and really come to know it and find a way to use the work they have done here to contribute to that body of literature.

Reviewer #3: Please use the space provided to explain your answers to the questions above. You may also include additional comments for the author, including concerns about dual publication, research ethics, or publication ethics. (Please upload your review as an attachment if it exceeds 20,000 characters) (Limit 200 to 20000 Characters)

This paper discusses the use of mathematical models based in epidemiological research to assess the spread of health information within populations, an important question and methodological application. While the authors have collected relevant information and conducted an interesting experiment, I think that the paper requires significant organizational revision to more clearly make the authors’ points and to strengthen its utility to readers. I have suggested a few revisions and asked several questions below.

1. Since this is a research article, I believe the introduction section could be greatly condensed and would be strengthened by focusing on providing context relevant to the key comparison: SIR vs SEIR models and a brief summary of the use of mathematical modelling in studying the dynamics of misinformation. It would also help to have further headings to show your details. It is reasonable to assume readers have some background in mathematical modelling or, if not, would consult referenced articles, rather than including the level of detail here and particularly the comparison of many model types not used in the experiment.

2. Several of the references in the article should be revisited. Specifically, citation 52 references a conference abstract when describing a shooting incident at a pizza shop; citing a news report or other write up of the incident directly would be preferable. The authors also reference several pre-prints, which in my experience are not usually appropriate for reference in a research article; for reference 17, for example, there are other peer-reviewed papers describing COVID-19 spread and models. Finally, the reference type changes through the presentation (some numbers, some names), and several references (for example, Z. Wang et al. 2017 on page 19) are not included in the reference list at all.

3. Tools and techniques: Again, please consider condensing the text and using references to allow readers to connect to further materials on the methods as needed.

4. Findings and results: unless otherwise stated by the editor, many of the steps around data cleaning could be removed from the main text and moved to supplemental materials unless critical for comprehension (for example, the level of detail around ‘tweet created’ might be replaced with a few sentences).

5. Note around the correlation: I think it’s a nice visualization. I’d note that correlation isn’t scored 0.83 out of 1, but rather on the scale from -1 to 1 (negative correlation is observed in the figure). It would be strengthened in interpretation with more detailed titles or a table below reminding the audience what each of the parameters are, as the names alone are hard to interpret.

6. Figure 10 is also helpful; could you please repeat the same visualizations and tables for both SIR and SEIR models? I think it would also be very helpful to have a table stating where the model parameters are generated from the dataset (see this paper for an example: https://www.sciencedirect.com/science/article/pii/S2666818123001225)

7. Machine learning: I don’t follow the final use of the mathematical models in the machine learning application. Were both or only one model used? If so, which is used in the performance metrics? Could you please also be more specific as to how this affects your ultimate model choice?

8. Model comparison: the model comparison discussion does not seem to relate the results of the experiment, but rather more general characteristics. Could you bring your results in more? Similarly, in model assumptions, consider breaking up this long paragraph into sections or using headings to more clearly convey your findings.

9. Practical implications: It’s stated that the models demonstrate that people can overcome misinformation, yet the Fig 10 shows a large infected group at the end?

10. In your conclusions and throughout the discussion, consider citing other articles to help contextualize your findings. Overall, I think with the condensing of some of the text, particularly early on, you would have room to share more findings from the experiment and especially the comparison.

**********

what does this mean?). If published, this will include your full peer review and any attached files.

**Do you want your identity to be public for this peer review?**  If you choose “no”, your identity will remain anonymous but your review may still be made public.

For information about this choice, including consent withdrawal, please see our Privacy Policy

Reviewer #1: No

Reviewer #2: **Yes:**  Sagar Kumar

Reviewer #3: No

**********

**Figure resubmission:**

**Reproducibility:**

To enhance the reproducibility of your results, we recommend that authors of applicable studies deposit laboratory protocols in protocols.io, where a protocol can be assigned its own identifier (DOI) such that it can be cited independently in the future. Additionally, PLOS ONE offers an option to publish peer-reviewed clinical study protocols. Read more information on sharing protocols at https://plos.org/protocols?utm_medium=editorial-email&utm_source=authorletters&utm_campaign=protocols

# Author comment: Author response to Decision Letter 1

**DOI:** 10.1371/journal.pdig.0001772.r002

24 Nov 2025

Attachment Submitted filename: REBUTTAL TO REVIEWER COMMENTS_NOV 2025.docx

# Peer review: Decision Letter 1

**DOI:** 10.1371/journal.pdig.0001772.r003

**Authors:** Leo Anthony Celi

19 Feb 2026

Response to ReviewersRevised Manuscript with Track ChangesManuscript**Journal Requirements:** **Additional Editor Comments (if provided):** **Reviewers' Comments:**

**Comments to the Author**

Reviewer #1: (No Response)

Reviewer #3: (No Response)

**********

publication criteria?

Reviewer #1: No

Reviewer #3: Partly

**********

3. Has the statistical analysis been performed appropriately and rigorously?

Reviewer #1: I don't know

Reviewer #3: I don't know

**********

4. Have the authors made all data underlying the findings in their manuscript fully available (please refer to the Data Availability Statement at the start of the manuscript PDF file)?

The PLOS Data policy

Reviewer #1: Yes

Reviewer #3: Yes

**********

5. Is the manuscript presented in an intelligible fashion and written in standard English?

Reviewer #1: Yes

Reviewer #3: Yes

**********

Reviewer #1: This is a much improved manuscript, and I commend the authors for their immense amount of work. However, I still have some major concerns, and some smaller ones as well.

My biggest concern is that my qualms about the Twitter data remained unaddressed. Despite raising a number of concerns about its use in my review, the only action taken was to cite the Kaggle dataset. The rest of my comment - copied below for a reminder - remained completely unaddressed. I still believe the Kaggle dataset to be wholly inappropriate for this analysis due to limitations in its size and scope and timeline. I encouraged the authors to discuss this when introducing the dataset, and framing it as a proof of concept work, or collect their own data set for a more robust analysis.

My previous review:

"The Kaggle dataset is not well explained, defined or cited - in fact, it isn’t cited at all in the references. There is simply a URL in the methods section! That is not adequate, and borders on lack of attribution. Given that the authors did not collect these data, I need many more details about their provenance. Can you put this dataset in the context of mpox? How were the variables collected? Are they representative of all tweets? From a quick look on the

Kaggle website, it looks like there are only data from July and August, and based on Figure 8, it looks like there are only 4 days of data, which is much too limited for this type of analysis."

I think, at very minimum, this dataset needs to be clearly described, including its provenance, fields collected and why, and the dates of inclusion, and in the limitations discuss much more in depth how this dataset was curated, what it's general purpose is (for educational exercises), and whether four days of data and ~5000 tweets are really representative of misinformation spread. In fact, in looking at figure 3, the vast majority of these tweets were generated on a single day - July 9th, 2022. That is quite concerning to me as there is really very little representativeness from a single data point. I understand that the idea is that this was a big surge in data from the earlier data points, but it is not clear from this research nor the Kaggle website whether this is all tweets over this period or why this period was chosen, both of which feel necessary to drive the results and conclusions of this paper. Noting this, many of the conclusions drawn about the interventions and possible mitigating impacts feel tenuous as this does not feel like a representative analysis to me.

My second major comment is that there was no appendix included with this resubmission. The authors mentioned moving many details to the appendix, but there was none included for me to review. In my next review, I would like to see this appendix and review it in depth as well.

In addition to these two major comments, I have a few smaller comments as well.

First, Figure 1 is a screenshot of the Kaggle dataset, and is inappropriate for inclusion in this analysis without redoing the table yourself. It is not cited, there is no evidence that the dataset authors allowed for this replication, and it really does not add any value beyond what I could learn by simply following the URL in the reference.

Second, all exploratory data analyses should be simply summarized and moved to an appendix, in particular the correlation figure and temporal figure. In fact, I have concerns about Figure 3 in general, how it implies a linear trend overtime which is not supported by the data. It does not, in fact, look like an epidemic curve, because there is no data in between July 9th and 13th on which to make that conclusion, and a simple linear interpolation is used instead.

Reviewer #3: Thank you to the authors for their thorough revision of the manuscript; it has a much clearer focus and is easier to follow than the previous draft with lots of well-documented references. I have a few additional questions and recommendations:

• In the typical background/methods/results/discussion layout, interpretation of the results in a broader context belongs in the discussion; I suggest moving sentences such as ‘These patterns align with established models of social media engagement cascades [8]’ and ‘However, the network structure did not reveal novel causal relationships beyond those expected from prior literature, suggesting that Bayesian networks may have limited utility for causal discovery in this context without experimental manipulation [37].’ to the discussion section.

• Thank you for including citations throughout the paper as suggested by the reviewers; in the results section, it’s not necessary to include citations for statements about the work completed within the paper and I would suggest you remove most of them (for example: ‘For example, unverified users show higher misinformation probability compared to verified accounts [31], and tweets with anomalously high retweet counts relative to other engagement metrics exhibit elevated misinformation risk [9].’ – no need for citations here).

• In the SIR models, it appears in 5 and 7 days that there is no additional depletion of susceptible although there are infectious people in the population; can you comment on this further? The authors state “Critically, the infectious compartment (I) exhibited the expected single-peaked trajectory: rising rapidly during the outbreak’s growth phase, reaching a maximum, then declining as the rate of new infections fell below the recovery rate”: there is no visible decline in the number of infected in any of the SIR visuals.

• I was unclear why the authors didn’t state the specific model parameters used (specifically the β, γ and σ); please see an example of how parameters were laid out in a recent modelling paper: https://pubmed.ncbi.nlm.nih.gov/37764955/. In particular, the recovery rate is critical to know as the time scale studied is very short (e.g., is a person recovered only 1 day after sharing a misinformation tweet?) I see the authors mention 5 hours, but wasn’t clear if that was for the recovery rate or the timing for someone to be still considered infectious.

• The above point on parameters is especially important for the SEIR model – it appears as though almost the entire population is immediately exposed; could you comment more on this assumption, and uncertainty there?

• The dataset used includes misinformation/other categories and misinformation/good information categorizations. Why did the authors chose misinformation/other vs misinformation/good? Presumably, users are exposed to of both ‘good information’ and ‘misinformation’, contrasting somewhat what we might expect with a disease where people are exposed or unexposed; it’d be helpful if the authors could comment on the implications for modelling.

• Was there a specific reason why the authors didn’t do a 1/3/5/7 day analysis for the SEIR model compared to the SIR model, or why there have different time scales?

• While I appreciate the authors’ attention to detail, the length of the conclusion relative to the content of the paper is a bit long; given that the conclusions are mostly stated in the discussion section, I would recommend this be shortened to one or two paragraphs maximum.

**********

what does this mean?). If published, this will include your full peer review and any attached files.

**Do you want your identity to be public for this peer review?**  If you choose “no”, your identity will remain anonymous but your review may still be made public.

For information about this choice, including consent withdrawal, please see our Privacy Policy

Reviewer #1: None

Reviewer #3: No

**********

**Figure resubmission:**

**Reproducibility:**

To enhance the reproducibility of your results, we recommend that authors of applicable studies deposit laboratory protocols in protocols.io, where a protocol can be assigned its own identifier (DOI) such that it can be cited independently in the future. Additionally, PLOS ONE offers an option to publish peer-reviewed clinical study protocols. Read more information on sharing protocols at https://plos.org/protocols?utm_medium=editorial-email&utm_source=authorletters&utm_campaign=protocols

# Author comment: Author response to Decision Letter 2

**DOI:** 10.1371/journal.pdig.0001772.r004

10 Apr 2026

Attachment Submitted filename: Response_to_Reviewers_09042026.pdf

# Peer review: Decision Letter 2

**DOI:** 10.1371/journal.pdig.0001772.r005

**Authors:** Leo Anthony Celi

11 May 2026

Response to Reviewers Revised Manuscript with Track Changes Manuscript **Journal Requirements:**

1. Some material included in your submission may be copyrighted. According to PLOS’s copyright policy, authors who use figures or other material (e.g., graphics, clipart, maps) from another author or copyright holder must demonstrate or obtain permission to publish this material under the Creative Commons Attribution 4.0 International (CC BY 4.0) License used by PLOS journals. Please closely review the details of PLOS’s copyright requirements here: PLOS Licenses and Copyright. If you need to request permissions from a copyright holder, you may use PLOS's Copyright Content Permission form.

Please respond directly to this email or email the journal office and provide any known details concerning your material's license terms and permissions required for reuse, even if you have not yet obtained copyright permissions or are unsure of your material's copyright compatibility.

Potential Copyright Issues:

a. Figures 10, 11: Please confirm whether you drew the images / clip-art within the figure panels by hand. If you did not draw the images, please provide (a) a link to the source of the images or icons and their license / terms of use; or (b) written permission from the copyright holder to publish the images or icons under our CC-BY 4.0 license. Alternatively, you may replace the images with open source alternatives. See these open source resources you may use to replace images / clip-art:

- https://commons.wikimedia.org

- https://openclipart.org/

2. We ask that a manuscript source file is provided at Revision. Please upload your manuscript file as a .doc, .docx, .rtf or .tex.

3. Please upload separate figure files in .tif or .eps format. Also, remove the figures from your manuscript file but keep the legends.

For more information about figure files please see our guidelines:

https://journals.plos.org/digitalhealth/s/figures

https://journals.plos.org/digitalhealth/s/figures#loc-file-requirements

4. Please provide an Author Summary. This should appear in your manuscript between the Abstract (if applicable) and the Introduction, and should be 150–200 words long. The aim should be to make your findings accessible to a wide audience that includes both scientists and non-scientists. Sample summaries can be found on our website under Submission Guidelines:

https://journals.plos.org/digitalhealth/s/submission-guidelines#loc-parts-of-a-submission

**Additional Editor Comments (if provided):** **Reviewers' Comments:**

**Comments to the Author**

Reviewer #1: All comments have been addressed

**********

publication criteria?

Reviewer #1: Yes

**********

3. Has the statistical analysis been performed appropriately and rigorously?

Reviewer #1: Yes

**********

4. Have the authors made all data underlying the findings in their manuscript fully available (please refer to the Data Availability Statement at the start of the manuscript PDF file)?

The PLOS Data policy

Reviewer #1: Yes

**********

5. Is the manuscript presented in an intelligible fashion and written in standard English?

Reviewer #1: Yes

**********

**Reviewer #1:**  This is a much improved manuscript and I appreciate all of the explicit details about the provenance of the data source and its limitations in your analysis. I have a few small further recommendations:

At the top of page 5, you write 'Consistent with reviewer guidance, we describe its provenance,

fields and temporal scope in detail and explicitly discuss the implications

of its limitations for our findings.'. This is quite non-standard for a journal article and I believe should be removed and just explained that the provenence and scope are described in the following section.

Similarly, on page 10, there is no need to describe that the "epidemic curve" appearance was an artifact. Removing the mention of the "epidemic curve" appearance altogether is preferrable, and keeping your reply to me is adequate.

Again, on page 21, there is no need to mention that you "now" highlight the limitation of the mpox kaggle dataset. Just jump straight into the limitations.

Basically, in sum, all of the parts of the manuscript that are pointed replies to the reviewer should be removed. Those belong in the response to reviewers. Making the requested changes and additions is enough for the manuscript itself without being explicit as to why the changes were made.

**********

what does this mean?). If published, this will include your full peer review and any attached files.

**Do you want your identity to be public for this peer review?**  If you choose “no”, your identity will remain anonymous but your review may still be made public.

For information about this choice, including consent withdrawal, please see our Privacy Policy

Reviewer #1: No

**********

**Figure resubmission:**

**Reproducibility:**

To enhance the reproducibility of your results, we recommend that authors of applicable studies deposit laboratory protocols in protocols.io, where a protocol can be assigned its own identifier (DOI) such that it can be cited independently in the future. Additionally, PLOS ONE offers an option to publish peer-reviewed clinical study protocols. Read more information on sharing protocols at https://plos.org/protocols?utm_medium=editorial-email&utm_source=authorletters&utm_campaign=protocols

# Author comment: Author response to Decision Letter 3

**DOI:** 10.1371/journal.pdig.0001772.r006

7 Jun 2026

Attachment Submitted filename: Response to Reviewers_05062026.docx

# Peer review: Decision Letter 3

**DOI:** 10.1371/journal.pdig.0001772.r007

**Authors:** Leo Anthony Celi

6 Jul 2026

Response to ReviewersRevised Manuscript with Track ChangesManuscript**Journal Requirements:** **Additional Editor Comments (if provided):** **Reviewers' Comments:**

**Comments to the Author**

Reviewer #1: (No Response)

**********

publication criteria?

Reviewer #1: Yes

**********

3. Has the statistical analysis been performed appropriately and rigorously?

Reviewer #1: Yes

**********

4. Have the authors made all data underlying the findings in their manuscript fully available (please refer to the Data Availability Statement at the start of the manuscript PDF file)?

The PLOS Data policy

Reviewer #1: Yes

**********

5. Is the manuscript presented in an intelligible fashion and written in standard English?

Reviewer #1: Yes

**********

Reviewer #1: I thank the authors again for their review. I identified a few small areas where the replies to reviewers or language around the review process were not fully omitted from the manuscript. After these are addressed, all of my feedback will have been thoroughly incorporated.

1) The caption of Table 2 still says "Reporting follows the layout suggested by the reviewer [33]."

2) The caption of Figure 3 retains the following text: "Why E rises so rapidly to encompass much of the population.

A reviewer noted that, with the parameters in Table 2, the SEIR simulation moves nearly the entire population into the exposed compartment within a short interval, which appears unrealistic."

3) Athough the language in the main text about appendix S2 has been modified, the S2 appendix itself still has the following language: "S2 Appendix: Exploratory Data Analysis and Matched-Horizon SEIR Runs. This appendix collects the exploratory figures that were previously embedded in the main text, together with the additional 1/3/5/7-day SEIR runs requested by Reviewer 3."

**********

what does this mean?). If published, this will include your full peer review and any attached files.

**Do you want your identity to be public for this peer review?**  If you choose “no”, your identity will remain anonymous but your review may still be made public.

For information about this choice, including consent withdrawal, please see our Privacy Policy

Reviewer #1: No

**********

**Figure resubmission:**

**Reproducibility:**

To enhance the reproducibility of your results, we recommend that authors of applicable studies deposit laboratory protocols in protocols.io, where a protocol can be assigned its own identifier (DOI) such that it can be cited independently in the future. Additionally, PLOS ONE offers an option to publish peer-reviewed clinical study protocols. Read more information on sharing protocols at https://plos.org/protocols?utm_medium=editorial-email&utm_source=authorletters&utm_campaign=protocols

# Author comment: Author response to Decision Letter 4

**DOI:** 10.1371/journal.pdig.0001772.r008

12 Aug 2026

Attachment Submitted filename: Response to Reviewers_.docx

# Peer review: Decision Letter 4

**DOI:** 10.1371/journal.pdig.0001772.r009

**Authors:** Leo Anthony Celi

24 Sep 2026

Epidemiological Models in Health Misinformation Propagation of MPox Conversations

PDIG-D-24-00603R4

Dear Dr Ajao,

We are pleased to inform you that your manuscript 'Epidemiological Models in Health Misinformation Propagation of MPox Conversations' has been provisionally accepted for publication in PLOS Digital Health.

Before your manuscript can be formally accepted you will need to complete some formatting changes, which you will receive in a follow-up email from a member of our team.

Please note that your manuscript will not be scheduled for publication until you have made the required changes, so a swift response is appreciated.

IMPORTANT: The editorial review process is now complete. PLOS will only permit corrections to spelling, formatting or significant scientific errors from this point onwards. Requests for major changes, or any which affect the scientific understanding of your work, will cause delays to the publication date of your manuscript.

If your institution or institutions have a press office, please notify them about your upcoming paper to help maximize its impact. If they'll be preparing press materials, please inform our press team as soon as possible -- no later than 48 hours after receiving the formal acceptance. Your manuscript will remain under strict press embargo until 2 pm Eastern Time on the date of publication. For more information, please contact digitalhealth@plos.org.

Thank you again for supporting Open Access publishing; we are looking forward to publishing your work in PLOS Digital Health.

Best regards,

Leo Anthony Celi, MD MS MPH

Editor-In-Chief

PLOS Digital Health

***********************************************************

**Additional Editor Comments (if provided):**

**Reviewer Comments (if any, and for reference):**

Reviewer's Responses to Questions

**Comments to the Author**

Reviewer #1: All comments have been addressed

**********

publication criteria?

Reviewer #1: Yes

**********

3. Has the statistical analysis been performed appropriately and rigorously?

Reviewer #1: Yes

**********

4. Have the authors made all data underlying the findings in their manuscript fully available (please refer to the Data Availability Statement at the start of the manuscript PDF file)?

The PLOS Data policy

Reviewer #1: (No Response)

**********

5. Is the manuscript presented in an intelligible fashion and written in standard English?

Reviewer #1: Yes

**********

Reviewer #1: Thank you for revising the comments about "responding to reviewer". All of my comments and concerns have been addressed.

**********

what does this mean?). If published, this will include your full peer review and any attached files.

**Do you want your identity to be public for this peer review?**  If you choose “no”, your identity will remain anonymous but your review may still be made public.

For information about this choice, including consent withdrawal, please see our Privacy Policy

Reviewer #1: No

**********
